# Inducing incentive sensitization of exercise reinforcement among adults who do not regularly exercise—A randomized controlled trial

**DOI:** 10.1371/journal.pone.0216355

**Published:** 2019-05-07

**Authors:** Kyle D. Flack, Kelsey Elise Ufholz, LuAnn Johnson, James N. Roemmich

**Affiliations:** 1 Department of Dietetics and Human Nutrition, University of Kentucky, Lexington, Kentucky, United States of America; 2 USDA, Agricultural Research Service, Grand Forks Human Nutrition Research Center, Grand Forks, ND, United States of America; Vanderbilt University, UNITED STATES

## Abstract

**Background:**

Increasing exercise reinforcement, or decreasing sedentary reinforcement, may reduce sedentary activity and promote habitual exercise. Repeated exposures to a reinforcer may increase its reinforcing value (i.e., incentive sensitization). It is not yet known whether incentive sensitization occurs for exercise or factors associated with incentive sensitization for exercise reinforcement. The purpose was to determine whether exercise exposures increase exercise reinforcement relative to a sedentary alternative and whether this sensitization of exercise reinforcement would alter physical or sedentary behavior. This work also determined whether exercise dose, intensity, and preference and tolerance for exercise intensity were associated with incentive sensitization of exercise.

**Methods:**

104 sedentary men and women were randomized to exercise training groups with 89 completing the study. Groups included exercise exposures of 150 (n = 35) or 300 kcal/session (n = 34), 3 sessions/week for 6 weeks, or a non-exercise control group (n = 35). Assessments for exercise and sedentary behavior reinforcement (primary dependent variables) and activity and tolerance for exercise intensity were performed at baseline (week 0), post training (week 6), and post washout (week 10).

**Results:**

The control group reduced (P = 0.022) relative reinforcing value of exercise, such that the 150 kcal group had a greater relative reinforcing value of exercise after the exercise treatment 150 kcal: 0.69 ± 0.07 to 0.74 ± 0.07; 300 kcal: 0.72 ± 0.07 to 0.63 ± 0.08, control: 0.72 ± 0.07 to 0.57 ± 0.08 mean ± SE. Increases in tolerance for exercise intensity discomfort were associated with increases in relative reinforcing value of exercise. Sedentary behavior reinforcement decreased in both exercise groups (150 kcal: 5.4 ± 4.3 to 1.8 ± 1.3; 300 kcal: 5.4 ± 4.3 to 3.1 ± 2.4, P<0.05), but remained unchanged in the control group (5.1 ± 4.0 to 6.1 ± 4.9, P>0.05). Sedentary activity decreased baseline to post-training in the 300 kcal group (546.5 ± 10.7 to 503.8 ± 11.8 minutes, P<0.01).

**Conclusion:**

Small amounts of regular exercise may reduce the reinforcing value sedentary behavior. The process of incentive sensitization of exercise may include reducing the reinforcing value of competing sedentary activities. Developing tolerance to exercise discomfort of exercise may be critical to increasing exercise reinforcement.

## Introduction

The CDC *Physical Activity Guidelines for Americans* and their inclusion in the Dietary Guidelines for Americans (DGA) provide evidence-based advice to promote health and to reduce risk for chronic diseases through physical activity (PA) [1, 2]. However, only one in four Americans report engaging in any leisure time PA [3]. The low adherence of most Americans to PA recommendations underscores the need to understand how to effectively make PA a habit. An ideal product would be exercise programs that simultaneously increase both fitness and the motivating value of being physically active, such that initiation of a regular exercise regimen also promotes long-term PA adherence.

The reinforcing aspects of behaviors are a product of the central dopamine system [4]. Exercise elicits central dopamine release and is considered a reinforcing behavior, with exercise dependency demonstrated in both humans [5–8] and rodents [8–11]. Cross-sectional data have established that adults who find aerobic exercise highly reinforcing are more likely to meet PA guidelines for aerobic exercise while those who find resistance-type exercise more reinforcing are more likely to meet PA guidelines for both muscle-strengthening and aerobic exercise [12], demonstrating the reinforcing value of exercise as a prime determinant in the choice to exercise [13]. Importantly, in adults, the reinforcing value of exercise behaviors influences the choice to be active separate from the hedonic response, or liking, of exercise [14]. Liking is influenced by the central opioid system whereas behavioral reinforcement, as mentioned, is controlled by central dopamine signaling [15, 16]. Exercise may serve as an alternative reinforcer by producing physiological changes in the central dopamine system that changes the reinforcing value of other behaviors such as drug abuse [17] and perhaps also sedentary behavior. The process of incentive sensitization of exercise may include reducing the reinforcing value of competing sedentary activities. Increasing the reinforcing value of exercise may be vital for physical activity guideline adherence and reducing sedentary behavior.

Increasing the reinforcing value of a stimulus, termed “incentive sensitization”, was originally proposed to explain drug addiction [18]. Incentive sensitization is realized through repeated exposures that produce neuroadaptations that increase the craving of the stimulus [19]. The extent to which exercise reinforcement can be increased has not yet been studied, although biologically plausible, as exercise elicits a dopamine response similar to other reinforcing behaviors such as drug abuse, gambling, and eating [5–11, 20, 21]. The development of sensitization of drug abuse is dose-dependent, with moderately high doses increasing reinforcement more than either low or very high doses [22]. However, there are not yet any data on what may be considered a low, moderate, or high ‘dose’ of exercise when the aim is to produce incentive sensitization of exercise reinforcement. Understanding the influence of various parameters of exercise on incentive sensitization would yield new information that would help individuals adhere to PA recommendations. As a first step in the research area, the present study aimed to determine whether incentive sensitization occurred for exercise and was dependent on exercise dose. The current study defines exercise “dose” as the amount of energy expired during each session, as the treatment groups consisted of 150, 300, and 0 kcal (0 kcal served as control group).

Another factor that may be associated with exercise reinforcement is the preference for and/or tolerance to the unpleasant aspects of exercise intensity [12, 23, 24]. Unpleasant aspects of intense exercise such as muscle pain, fatigue, and breathing hard can elicit lower ratings of pleasure and affect [12, 23, 24]. This is especially observed in novice exercisers and overweight individuals [25], which may make it difficult for these individuals to adhere to an exercise program. Indeed, individuals who have greater tolerance for these unpleasant aspects of exercise are more likely to meet PA guidelines [12]. Moreover, a greater tolerance for exercise intensity discomfort is positively associated with the reinforcing value of resistance exercise [14], pointing to the possibility that the ability to tolerate the unpleasant aspects of exercise and exercise reinforcement are connected. Whether tolerance for exercise discomfort can be increased with repeated exposures to exercise is of both theoretical and practical interest. Longitudinal data demonstrating that increases in tolerance for exercise discomfort after exercise exposures are associated with increases in exercise reinforcement would provide strong evidence for the important role that developing tolerance for exercise intensity may play in exercise reinforcement. Thus, the primary objectives of the current study were to determine whether exercise reinforcement could be increased, or sedentary behavior reinforcement decreased, with exercise exposures in adults who were not engaging in a regular exercise program, whether incentive sensitization of exercise is dependent on the amount of exercise completed during the exposures, and whether incentive sensitization of exercise increases usual PA or decreases time spent in sedentary activities. Self-selected exercise training intensity during the exercise sessions and alterations in the preference and the tolerance to exercise discomfort were assessed as correlates of incentive sensitization of exercise. It was hypothesized that exercise reinforcement is dose dependent; groups exercising at 150 and 300 kcal per session would increase their reinforcing value of exercise more than the control (0 kcal per day) group, with the 300 kcal per session experiencing the greatest increase. It was also hypothesized that increases in preference and tolerance would be associated with increased incentive sensitization.

## Materials and methods

### Participants and study design

Power analysis: Power was based on demonstrating initial increases in RRV of physical activity during the 6 wk treatment period. Assuming an α = 0.01 (two sided) and a between-subject SD = 10, 30 subjects/group were needed to have 90% power to detect a mean difference in RRV of 7 units using paired t-tests (40% difference) between the 2 levels of physical activity treatment group at the end of the 6 wk treatment period. Previous studies [26, 27] have reported a between subject SD for the RRV of physical activity to range from 35% to 55% of the mean. A SD of 10 represents the upper point of this range. With a predicted attrition of 20%, the sample size was set at 35 to 36 per group.

**Fig 1:**
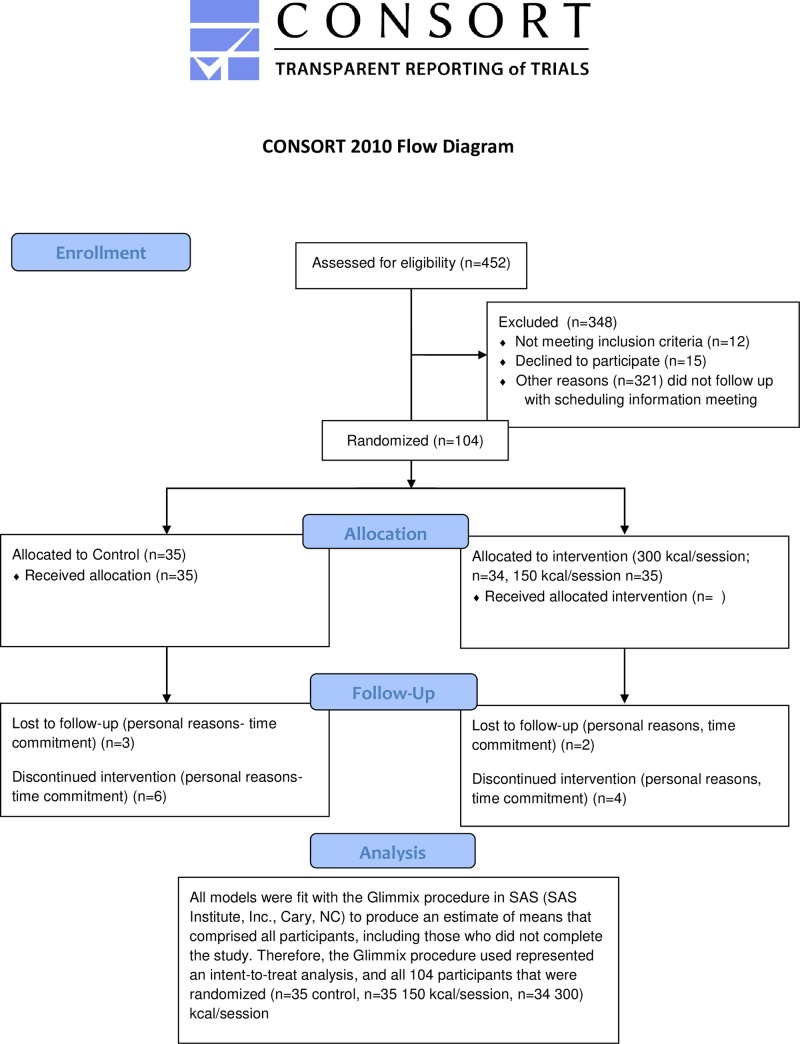
Consort flow diagram.

A total of 104 participants (86 female, 83%) age 18 to 49 years volunteered for the longitudinal, randomized, controlled trial. Of these, 89 participants completed the study (73 female), with 15 participants (14 female) voluntarily withdrawing citing personal reasons (Fig 1). Statistical analyses were performed on all 104 participants who were enrolled in the study. Participant age was capped at 49 years to reduce the risk of health problems compromising participant safety [28]. Participants were screened for not engaging in exercise more than one time per week over the past six months. This entry criterion was determined by a pre-enrollment interview where only those reporting not engaging in exercise, defined as planned or purposeful physical effort, completed to promote health and fitness, more than one day per week. Participants had a body mass index (BMI) ranging from 19–35 kg/m^2^ (Table 1). Recruitment began in the winter of 2015 and continued until recruitment goals were met (spring of 2017) in the greater Grand Forks, North Dakota metropolitan area. Participants were a sample who responded to recruitment media including printed brochures and fliers and online advertisements placed on the Grand Forks Human Nutrition Research Center website. All participants were non-smokers and healthy enough to participate in an exercise program assessed by the physical activity readiness questionnaire (PAR-Q). The study was approved by the University of North Dakota Institutional Review Board and registered with ClinicalTrials.gov, number NCT02444247.

**Table 1:** Baseline anthropometrics and physical activity of study participants randomized to an exercise intervention expending 150 or 300 kcal per exercise session, 3 days per week for 6-weeks, or to a non-exercise control group.

	150 kcal (n = 35)	300 kcal (n = 34)	Control (n = 35)
Age (years)	28.9 ± 10.8	27.1 ± 9.1	28.2 ± 9.7
Weight (kg)	73.4 ± 12.8	74.2 ± 15.4	72.1 ± 13.7
Height (cm)	168.9 ± 6.6	169.0 ± 8.1	167.4 ± 6.7
BMI (kg/m^2^)^1^	25.7 ± 4.3	25.8 ± 4.2	25.7 ± 4.3
MVPA (min/day)^2^	32.5 ± 22.1	27.7 ± 13.9	29.9 ± 25.4
Sedentary (min/day)^3^	547.7 ± 81.6	539.1 ± 80.6	546.7 ± 78.7

Data are mean ± SD

^1^BMI: body mass index

^2^MVPA: Minutes/day of moderate-to-vigorous physical activity

^3^Sedentary: minutes/day spent in sedentary activity

During visit one, after having the study explained and providing written informed consent, participants were measured for anthropometrics (height, weight) and given an ActiGraph accelerometer (Pensacola, FL) to measure baseline usual PA. Participants wore the accelerometer for seven days before performing baseline assessments. During subsequent visits, participants completed assessments of exercise reinforcement and preference and tolerance for exercise intensity. Each measurement was assessed at baseline, after the 6-week prescribed-exercise intervention (post), and after a 4-week follow-up period during which no exercise was prescribed and participants were told they could be as active or inactive as they wished.

Upon completion of baseline assessments, participants were randomized into exercise treatment groups expending 150 or 300 kcal per exercise session, 3 days per week for 6 weeks, or a non-exercise control group. Participants were block randomized on baseline minutes of moderate to vigorous physical activity (MVPA), age, sex, and exercise reinforcement (RRV_exercise_, see exercise reinforcement test below). The random allocation sequence was generated using the Plan procedure in SAS. Participants were assigned in order of enrollment by the study statistician.

### Exercise intervention

Exercise dose can be manipulated by the length of the exercise program (number of weeks), frequency (sessions per week), duration of each session, or total energy expended per session (which is a function of duration of each session and exercise intensity). Groups in the current study expended 150 kcal or 300 kcal per session, 3 days per week for 6 weeks at a self-selected intensity in addition to the non-exercise control group. The current study manipulated the total energy expended of the exercise sessions, as opposed to time or intensity, to standardize the amount of work being done across participants. Differences in body mass would result in different amounts of energy being expended across participants if exercise sessions were prescribed based on time. Even within participants, standardizing exercise session duration could result in vastly different energy expenditure due to variations in intensity, that is, a 30-minute run would provide a greater dose of exercise than a 30-minute walk. The current study did not control for exercise intensity to give participants autonomy to self-select the intensity of their sessions, which is an important feature of developing intrinsic motivation for behavior change [29]. This was considered a low-dose intervention as both groups exercised below the recommended 150 min of MVPA per week [1, 2]. The 300 kcal/session group averaged just under 137 minutes of exercise per week (45.6 min/session) while the 150 kcal/session group averaged just under 80 minutes per week (26.5 min/session). These doses were chosen because the study aim was to increase exercise reinforcement. Prescribing exercise that met physical activity recommendations could have been too great of an exercise dose for previously non-exercising individuals resulting in an exercise program (sessions) that had low reinforcing value and produced low positive affect; resulting in reduced compliance and study retention and increased risk of injury. All these factors would have hampered the ability to study incentive sensitization of exercise [30, 31]. Aerobic exercise reinforcement is not likely to become sensitized when the exercise exposures are not reinforcing due to either a lack of autonomy over the mode or intensity of exercise, or the dose being too great.

To record and monitor the exercise sessions, participants were provided a SenseWear® and SenseWear + display® (BodyMedia, Pittsburg, PA) [32] that monitored and provided feedback regarding estimated exercise energy expenditure of each exercise bout. Participants were instructed to exercise until the SenseWear device indicated they had reached the assigned exercise energy expenditure for each session. Participants were provided no instruction regarding exercise intensity, only that it must be aerobic-type exercise (e.g., walking, running, biking, elliptical) and not to engage in any other exercise other than what they were prescribed (either 300 or 150 kcal per session, 3 days per week). The control group was instructed not to exercise or increase their PA over the 6-week intervention period. Participants in the 150 and 300 kcal groups were provided a 6-week pass to a local fitness center upon beginning the exercise intervention to assure access to exercise facilities, although they were free to exercise at home or outside if they wished. Participants returned to the laboratory every two weeks to download their SenseWear device to monitor protocol compliance, including the duration (minutes), exercise energy expenditure, and MET intensity (metabolic equivalents) of each exercise session. Participants slightly exceeded the 150 and 300 kcal targets (see Results). No participants were disqualified for non-compliance. Participants in the control group were provided a pass to the fitness center after the intervention.

## Assessments

### Anthropometric measures

Height was measured in triplicate to the nearest 0.1 cm using a stadiometer (Seca; Chino, CA). Body weight was measured using a calibrated digital scale (Fairbanks Scales- Model SCB-R9000-HS; MO) to the nearest 0.1 kg. Measures were completed with participants wearing either provided lab scrubs or light casual clothes (t-shirt, shorts) and not wearing shoes.

### Liking

Participants’ liking (hedonic value) of the exercise options (treadmill, elliptical, stationary bike) and sedentary alternatives (TV, video games, reading, puzzles/Sudoku) was assessed using a 10-point scale (1 = “do not like at all” and 10 = “like very much”). The most liked activity was used for the exercise reinforcement testing session (described below).

### Exercise reinforcement (primary outcome measure)

Exercise reinforcement can be measured by the amount of operant responding an individual is willing to complete to engage in exercise, in that if exercise is highly reinforcing, it will support more responding [33–35]. When measuring exercise reinforcement, the measurement environment usually includes a sedentary alternative as a response option so that the person is not responding for access to exercise out of boredom and to mimic most real-world scenarios where the choice to exercise is made over a sedentary alternative. The reinforcing value (amount of responding) of one behavior is determined relative to the other and termed ‘relative reinforcing value’ (RRV) [12, 14, 36].

Participants’ RRV_exercise_ (specifically, aerobic-type exercise, bicycle ergometer, treadmill, or eliptical) was assessed against a sedentary alternative (reading magazines, playing word games, crossword puzzles, watching TV, playing video games). Two workstations were available for participants to complete the operant responding task (mouse button presses) for the behavior they desired. One station was a computer where the participant could earn points towards their most liked exercise activity while the other station was a computer that could be used to earn points toward their most liked sedentary alternative. Participants could switch between stations as much as they chose. The program presented a game that mimics a slot machine; a point is earned each time the shapes match. For every 5 points a session is completed and the participant receives 5 min of access to the reinforcer that was earned (either exercise or sedentary activity). The game is performed until the participant no longer wishes to work for access to either the exercise or sedentary activities. At first, points are delivered after every 4 presses, but then the schedule of reinforcement doubles (4, 8, 16, 32, […] 1024) each time 5 points are earned. For instance, the participant initially has to click the mouse button 4 times to earn each point for schedule 1. After the first 5 points are earned, schedule 1 is complete and the participant earns 5 minutes for exercise. Then 8 clicks are required to earn each of the next 5 points for schedule 2 before another 5 minutes of exercise is earned. Schedule 3 would require 16 clicks to earn one point, schedule 4 would require 32 clicks to earn one point and so on [36, 37]. The game ends when the participant no longer wishes to earn points (time) for exercise or the sedentary alternative. Upon completion, participants engaged in the activity for the time earned. The more reinforcing exercise or the sedentary behavior is, the more operant responding participants will do for access to these behaviors. Similar button pressing tasks are valid predictors of the RRV of physical versus sedentary activity and for determining the reinforcing value of food [33, 35, 38]. Participants self-selected the intensity level when performing any earned exercise time, which was typically a low to moderate steady-state intensity. These assessments took place in private laboratory space within a large exercise facility and the exercise facilities’ equipment was available for the subject to engage in the exercise that they had earned during the task. The last schedule completed for exercise and the sedentary alternative were assessed separately and termed Pmax of exercise (Pmax_exercise_), and Pmax of sedentary (Pmax_sed_). Units of Pmax were conceptualized as the number of clicks required to earn each point of the last schedule completed (i.e., 4, 8, 16, 32…). For instance, if a participant completed schedules 1, 2, and 3 for exercise and 1, 2, 3 and 4 for sedentary activities, this participant’s Pmax_exercise_ would be 16 and Pmax_sed_ would be 32. The greater the Pmax, the more “work” the participant was willing to do for the activity and thus the more reinforcing the activity is. RRV_exercise_ was defined as Pmax_exercise_ divided by the total number of schedules completed (Pmax_exercise_ plus Pmax_sed_) [39, 40].

### Physical activity and sedentary behavior (secondary outcome measures)

Habitual, free-living PA and sedentary behavior were measured using an ActiGraph accelerometer (GT3X+ model; Pensacola, Florida). Each participant wore the device for seven days on three occasions (prior to any baseline testing, immediately after completing the 6-week exercise intervention and assessments, and at week 10). Participants were instructed to wear the monitor at the hip using the provided belt during all waking hours except when bathing or swimming. Data were cleaned of non-wear time, defined as consecutive strings of zeros greater than 20 minutes. An epoch of 10 seconds was used for data collection as a shorter epoch is more suitable to reflect bout duration under free-living conditions where many bouts of sporadic PA last 30 seconds or less [41, 42]. These data were used to determine participants’ weekly minutes of MVPA as well as minutes of sedentary activity and light intensity activity using the Crouter et. al algorithm [43], and Freedson cut-points [44].

### Preference and tolerance for exercise intensity (secondary outcome measure)

The Preference for and Tolerance of the Intensity of Exercise Questionnaire (PRETIE-Q) [45, 46] was completed to measure how much a person tolerates and/or prefers the discomfort associated with intense exercise [46–48]. This was assessed by questionnaire during the initial screening/ consenting visit and on the final follow up visit separate from any bout of exercise. Preference and tolerance scores are associated with the frequency of participation in strenuous exercise and total leisure-time exercise [12], as well as being a strong predictor of PA behavior [45].

### Analytic plan

The outcome variables of Pmax_sed_, Pmax_exercise_, RRV_exercise_, and preference and tolerance for exercise intensity were assessed for differences between groups and across time (baseline, post training, and follow-up) and their interaction in an analysis of covariance (ANCOVA) using the corresponding baseline values as covariates. Each measure of PA (7-day totals of sedentary time, moderate, vigorous and MVPA) was analyzed using the corresponding baseline value as the covariate. Tukey contrasts were used to compare time points within each group and groups across each time point. Contrasts were also used to test whether for each group the rate of change across time was different from zero and if the rates of change differed between exercise groups, with Bonferroni adjustments for multiple comparisons. All models were fit with the Glimmix procedure in SAS (SAS Institute, Inc., Cary, NC) to produce an estimate of means that comprised all participants, including those who did not complete the study. Therefore, the Glimmix procedure analyses represented an intent-to-treat analysis, with scores from all participants randomized to an intervention included within the groups that they were assigned. Within the Glimmix procedure, the Gaussian distribution was used to model all variables except RRV_exercise_ which, because it is a ratio, was modeled using the beta distribution. Pearson correlation was used to test the associations between changes in RRV_exercise_ and PA, preference and tolerance, and the average intensity of exercise sessions during the intervention. Pmax_sed_ and Pmax_exercise_ were not normally distributed and thus log_2_ transformed. Correlation analysis was also performed to test if the MET intensity of training sessions or duration of training sessions was correlated to Pmax_sed_ or Pmax_exercise_. Separate ANCOVA analyses were performed assessing changes in RRV_exercise,_ Pmax_sed_, Pmax_exercise_ and PA using BMI and age as additional covariates to determine if weight status or age played a role in incentive sensitization for exercise reinforcement or changes in PA. A moderation analysis was performed to determine whether the average MET intensity of exercise during the intervention sessions moderated the association of change in tolerance for exercise discomfort with the change in RRV_exercise_.

## Results

**Fig 2:**
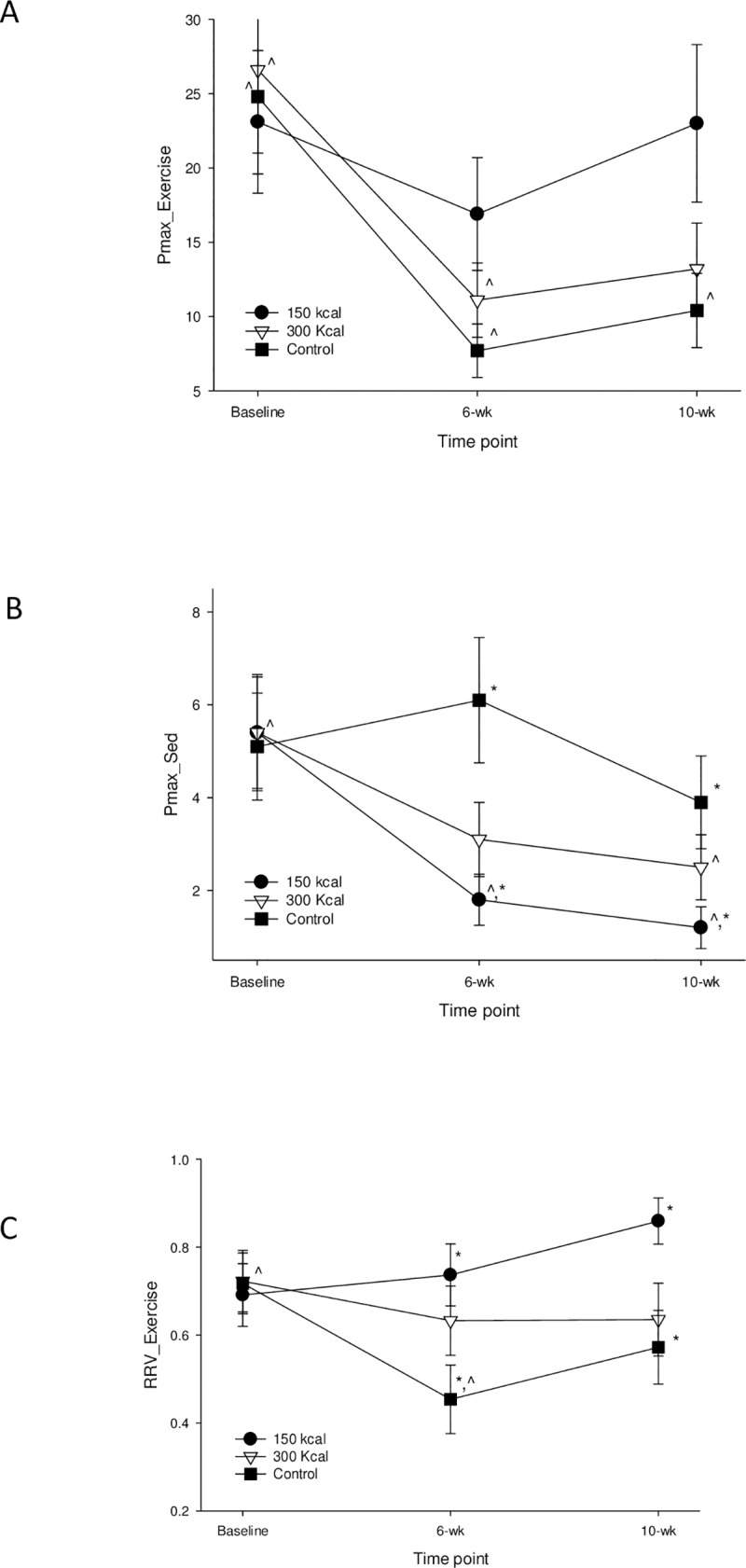
Changes in exercise and sedentary behavior reinforcement and relative reinforcing value of exercise over 10 weeks. Reinforcing value of exercise (Pmax of exercise, A), Pmax of sedentary activities (B), and relative reinforcing value of exercise (RRV_exercise_, C), at baseline, post-exercise intervention (6-week), and at the 4-week follow-up (10-week) among study participants enrolled in an exercise intervention expending either 150 or 300 kcal per exercise session, 3 days per week for 6-weeks, or in a non-exercise control group. * between group difference at a given time point (p < 0.05). ^ within groups change across time points p < 0.05. Values are mean ± SE.

During the intervention, participants in the 300 kcal group expended 335.6 ± 5.7 kcal per session whereas the 150 kcal group expended 182.0 ± 6.3 kcal (mean ± standard error, SE). The group by time interaction for Pmax_exercise_ was not significant (p = 0.23). Pmax_exercise_ decreased (P<0.0001) from baseline to post-training (6 weeks, P<0.0001) and from baseline to post-washout (10 weeks, P = 0.011). Simple effects analyses (Fig 2) showed that Pmax_exercise_ decreased (P = 0.013) from baseline to post-training in the 300 kcal group; and from baseline to post-training (P = 0.0004) and baseline to post-washout (P = 0.015) in the control group. There was a significant group by time interaction (P = 0.047) for Pmax_sed_ (Fig 2). Pmax_sed_ decreased in the 150 kcal group between baseline and post-training (6 weeks, P = 0.003) and between baseline and post-washout (10 weeks, p<0.0001), while the 300 kcal group decreased (p = 0.049) between baseline and post-washout. No changes were observed (P = 0.31) in the control group. The rate of change in Pmax_sed_ was negative (P≤0.048) from baseline to post-washout (10 weeks) for both exercise groups. Based on the changes in Pmax_exercise_ and Pmax_sed_ the treatment by phase interaction for RRV_exercise_ was P = 0.066. Examination of the simple effects analyses was informative as the control group reduced (P = 0.022) RRV_exercise_ between baseline and post-training (6 weeks) and the 150 kcal group had a greater RRV_exercise_ at the post-training (P = 0.035) and the post-washout (10 weeks, P = 0.018) phases compared to the control group (Fig 2). A treatment effect was observed (P = 0.048) for RRV_exercise_ in that the 150 kcal group had a greater (P = 0.037) RRV_exercise_ than the control group. Covarying for BMI or age did not influence the results presented above for Pmax_exercise_, Pmax_sed_, and RRV_exercise_. The average MET intensity of exercise during the exposure sessions did not moderate (P≥0.26) the association of change in tolerance for exercise discomfort on change in RRV_exercise_.

**Fig 3:**
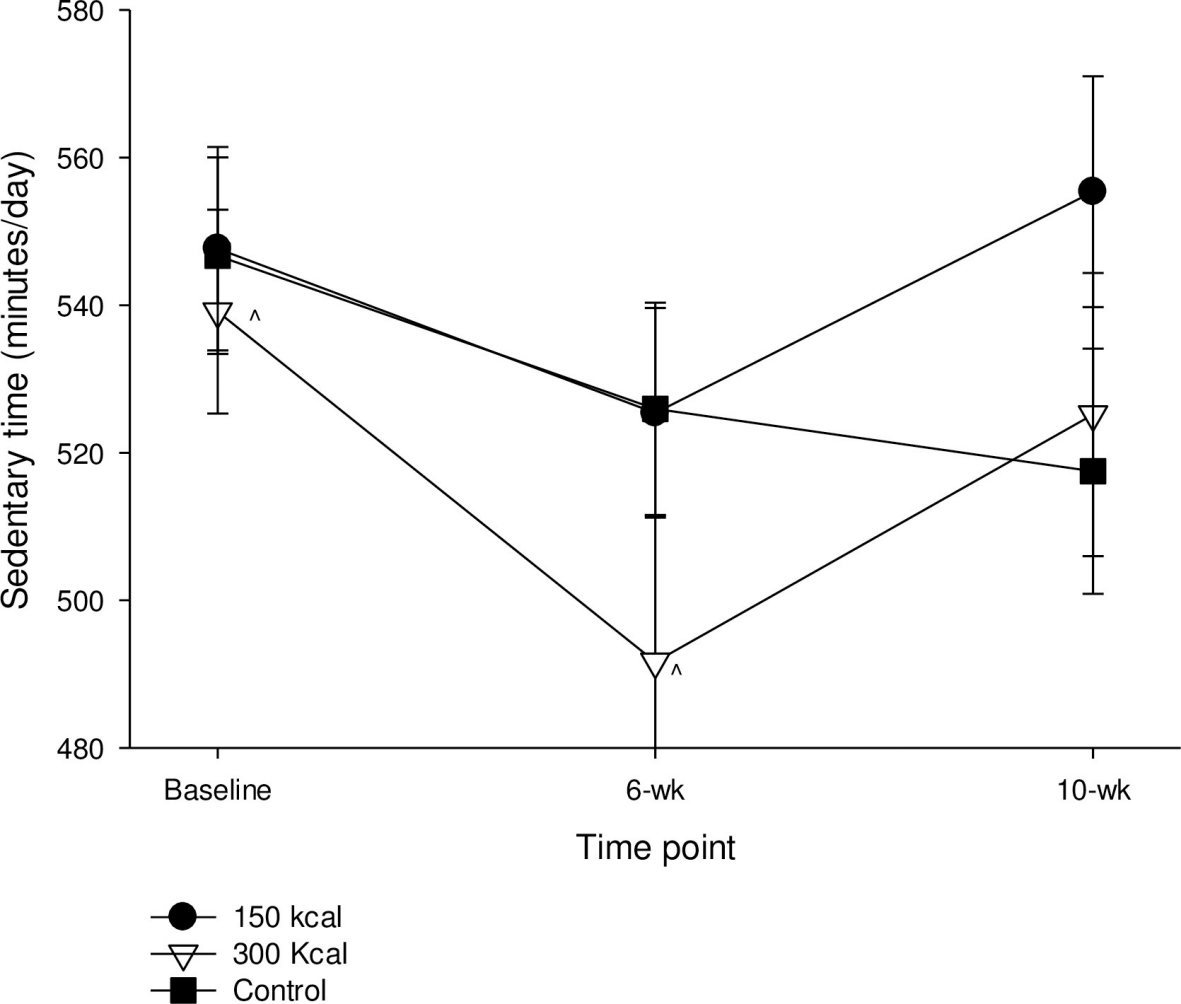
Time spent in sedentary activity assessed via accelerometry from baseline to post (6-week) and at 4-week follow-up. ^ indicates within groups change across time points p < 0.05. Values are means ± SE.

As shown in Fig 3, minutes of sedentary time decreased (P<0.01) baseline to post-training for the 300 kcal group. MVPA did not change across any time point for any group. The lack of change in MVPA in the 300 kcal group despite the decrease in sedentary time was due to the increase (P<0.05) in time spent in light PA (baseline: 215.0 ± 9.2, post training: 255.3 ± 10.5, post washout 267.7 ± 10.7). Covarying for BMI or age did not influence the results presented for MVPA. Univariate correlation analyses between changes in RRV_exercise_ and baseline MVPA (from baseline to 6 weeks, r = -0.08, and from baseline to 10 weeks, r = -0.15) were negligible to weak and produced no significant relationships (all P >0.12). Grouping participants into those who did or did not meet MVPA recommendations at baseline did not change the results and produced no group by time interactions for changes in RRV_exercise_ (P = 0.56), or Pmax_exercise_ (P = 0.82).

As shown in Table 2, tolerance for exercise discomfort increased between baseline and post-washout (10 weeks, P = 0.04 from completers analysis, P = 0.08 from intent to treat analysis). Neither preference (P>0.30) nor preference + tolerance for exercise intensity changed (P>0.10) across time or differed (P>0.40) between groups.

**Table 2:** Preference and tolerance of exercise intensity discomfort at baseline (week 0), post intervention (week-6) and 4 (week 10) of study participants enrolled in an exercise intervention expending either 150 or 300 kcal per exercise session, 3 days per week for 6-weeks, or in a non-exercise control group.

	150 kcal			300 kcal			Control		
	Baseline	Week 6	Week 10	Baseline	Week 6	Week 10	Baseline	Week 6	Week 10
Preference	25.3±1.1 [23.1, 27.5]	25.8±1.2 [23.5, 28.2]	25.5±1.2 [23.2, 27.8]	25.2±1.2 [22.9, 27.5]	25.5±1.2 [23.2, 27.8]	25.0±1.2 [22.6, 27.3]	23.3±1.2 [21.1, 25.6]	24.2±1.2 [21.9, 26.4]	23.6±1.2 [21.3, 25.8]
Tolerance*	22.3±0.9 [20.6, 24.0]	23.1±0.9 [21.2, 24.9]	23.5±0.9 [21.6, 25.3]	21.9±0.9 [20.2, 23.7]	23.8±0.9 [22.1, 25.6]	23.4±0.9 [21.6, 25.3]	23.0±0.9 [21.2, 24.7]	22.2±0.9 [20.5, 24.0]	23.5±0.9 [21.8, 25.3]
Preference + Tolerance	47.6±1.6 [44.4, 50.8]	49.0±1.7 [45.5, 52.4]	49.1±1.7 [45.7, 52.5]	47.1±1.7 [43.8, 50.4]	49.4±1.7 [46.1, 52.7]	48.5±1.7 [45.1, 51.8]	46.3±1.7 [43.0, 49.6]	46.4±1.7 [43.1, 49.7]	47.1±1.7 [43.8, 50.5]

Data are mean ± SE

95% CI shown in brackets

*Time effect between baseline and week 10 (P = 0.08 from intent to treat analysis, P = 0.04 from completers analysis

As shown in Table 3, changes in tolerance for exercise intensity and changes in preference + tolerance weakly correlated (P<0.01) with the change in RRV_exercise_. Also, the self-selected MET intensity of the exercise training sessions was weakly positively correlated (P<0.05) with changes in preference + tolerance for exercise intensity among participants assigned to the exercise intervention groups. When analyzing each group individually, the change in preference + tolerance was moderately correlated (r = 0.43, P<0.05) with MET intensity of the exercise sessions in the 150 kcal group. A moderate correlation was also observed between changes in tolerance and changes in RRV_exercise_ (r = 0.41, P<0.05) in the 150 kcal group.

**Table 3:** Simple (Pearson) correlations (r) between the changes in preference and tolerance for exercise intensity and the change in RRV_exercise_ from baseline to after 6-weeks of exercise training and correlations between the changes in preference and tolerance for exercise intensity and the average intensity of exercise training.

	Δ RRV_exercise_	Training intensity (Mets)
Δ Preference	0.13	0.22
Δ Tolerance	0.30**	0.22
Δ Preference + Tolerance	0.28**	0.29*

*P<0.05

**P<0.01

Analyses limited to those participants who completed either the 150 kcal/session or 300 kcal/session interventions, control group participants were not included.

In attempt to identify other aspects of the training sessions, aside from energy expended, that were associated with changes in Pmax of exercise and sedentary behaviors, average MET intensity of the exercise sessions or exercise duration were correlated to Pmax_sed,_ Pmax_exercise_, and RRV_exercise_. No significant correlations were found amongst these variables (all P>0.2).

## Discussion

The present study is the first RCT aimed at investigating whether a low-dose exercise program (i.e., below recommended minutes per week) could induce incentive sensitization of exercise reinforcement among adults who were engaging in no more than one day of exercise per week. Though there were no specific entry criteria for sedentary behavior, baseline accelerometer sedentary behavior of all the study groups exceeded usual adult values [49]. The present study also assessed whether factors including the self-selected intensity of the exercise sessions (exposures) and the change in tolerance to exercise intensity discomfort were associated with the incentive sensitization of exercise reinforcement.

A novel finding of the current study is that both the 150 kcal and 300 kcal exercise groups experienced a decrease in the reinforcing value of sedentary behaviors (Pmax_sed_, Fig 2). Exercise can reinforce operant responding in rodents [50] and as demonstrated in the present and previous [12,14] studies, exercise can serve as an alternative reinforcer to sedentary behavior. Moreover, in humans [51] and in rodents [17], even low amounts of exercise modify dopamine receptor binding in the mesolimbic pathway that reduce reinforcement of addictive drugs. Some sedentary behaviors mimic many of the qualities of addictive and binge-producing behaviors [52, 53]. Perhaps, even small amounts (150 kcal, 3 days/wk) of regular exercise in humans also alters central dopamine physiology that reduces the reinforcing value sedentary behavior. Thus, the process of increasing RRV_exercise_ may include reducing the reinforcing value of sedentary activities. The result would be to shift choice away from these highly reinforcing sedentary behaviors, as observed by the decrease in sedentary time among the intervention groups. Notably, a high amount of sedentary time was not required for acceptance into the present study, so the effect of repeated exposures of exercise on reductions in Pmax_sed_ and sedentary time may be even greater in studies that include participants with greater sedentary time than the current study population, especially non-work screen time, as an entry criterion.

The present study did not demonstrate incentive sensitization for exercise, that is, neither the Pmax_exercisen_ or RRV_exercise_ measures of exercise reinforcement increased over time. Understanding how to most effectively increase the reinforcing value of exercise will require additional research that tests different exercise session parameters (energy expended per session, duration of sessions, frequency of sessions, intensity of exercise, duration of intervention) and combinations of exercise parameters than those chosen for the current study. This is a new area of research, so the optimal parameters to induce incentive sensitization of exercise are not yet known. Based on the drug sensitization literature, sensitization occurs most readily after administration of moderately high doses [22], so we hypothesized that the 300 kcal per session treatment would cause greater sensitization than the 150 kcal per session treatment. However, it was unknown what constituted a “moderately high” dose of exercise for an individual who was not regularly exercising. From the current study, it seems that 6 weeks of exercise at a self-selected intensity 3 days per week expending at least 150 kcal is sufficient to decrease the reinforcing value of sedentary behaviors, but not to evoke greater exercise reinforcement. However, care must be taken in prescribing large doses of exercise to individuals who have not been regularly engaging in exercise. Similar to how excessive doses of drugs are not reinforcing but rather produce aversive effects [54], unfit, non-exercising adults would not likely find large doses of exercise reinforcing.

The lack of incentive sensitization was not likely an issue of the exercise being too “easy” or too similar to light activity as participants exercised at a mean intensity of 5.2 METs (the upper levels of moderate intensity). Rather, the exercise intervention may not have provided a great enough total volume (number of sessions per week, duration of intervention) to promote incentive sensitization. Modifying these variables of exercise dose and their impact on exercise reinforcement are all exciting possibilities for future research. By testing RRV_exercise_ and Pmax _exercise_ 6 weeks after commencing the exercise treatments, initial changes in these variables may not have been detected. RRV and Pmax_exercise_ may have increased during the initial few weeks and then declined through satiation or habituation from that point. Satiation or habituation of exercise reinforcement may be an especially relevant explanation for the reduction in Pmax_exercise_ in the 300 kcal group given the greater amount of exercise completed by those participants. However, notably, the simultaneous reduction in Pmax_sed_ allowed for a maintenance of RRV_exercise_ in the 300 kcal group. Thus, the current results provide initial evidence that an amount of exercise below that of current physical activity recommendations for adults may be a key factor in producing incentive sensitization of exercise in non-active adults. Additional research is needed to determine the optimal number of exposures and characteristics of those exposures needed to fully realize incentive sensitization for exercise.

At baseline, 70 of the 104 participants, accumulated nearly 30 minutes of MVPA per day. However, by screening interviews, it was assured that the study participants were not participating in active sports or structured exercise defined as purposeful physical activity undertaken with the goal to improve fitness. Therefore, the baseline MVPA was likely activities of daily living, recreational activity, and active transportation. The majority of participants were associated with a nearby University and walked across a sprawling campus to engage in classes and other student or staff functions. Only one participant met the VPA criterion of 75 minutes per week, which would be more indicative of exercise behaviors [1, 2] and removing this participant did not change the outcomes. Moreover, neither baseline MVPA nor total activity were correlated with the change in RRV_exercise_. Furthermore, changes in RRV_exercise_ or Pmax did not differ for participants meeting or not meeting PA recommendations at baseline. Thus, the lack of incentive sensitization for exercise reinforcement in our sample is not believed to be due to the study participants already habitually choosing to exercise over sedentary activities.

Unexpectedly, participants responded more for exercise behaviors than sedentary at baseline. This may have been due to the environment where the assessments took place. The RRV tests were performed at a laboratory space within a large fitness center, which may have inclined participants to select the exercise option, both out of curiosity and social desirability perceptions. Future research on environmental and social factors on exercise reinforcement may be an important area for future study.

Previous cross-sectional research [12] demonstrated that tolerance for exercise intensity discomfort is associated with RRV_exercise_. The current study extends these findings and provides stronger longitudinal evidence of this relationship by demonstrating that increases in tolerance to exercise intensity are associated with increases in RRV_exercise_. As such, developing greater preference and tolerance to exercise intensity discomfort may be important for incentive sensitization of exercise. Interestingly, the average intensity of the exercise sessions, which was self-selected, positively correlated with increases in preference and tolerance. This suggests that if exercise exposures are of sufficient intensity to induce greater discomfort, then they may help develop a preference and tolerance to the unpleasant aspects of exercise. The positive relationships between exercise training intensity and increases in preference and tolerance for exercise discomfort and between increases in preference and tolerance and RRV_exercise_ were only significant in the 150 kcal/session group. This suggests that exercise performed at greater intensities, even if it is of lower dose/duration, may be more effective for increasing preference and tolerance for exercise intensity. A small dose of exercise in non-exercising adults, a dose well below the physical activity recommendations for adults can produce alterations in psychological processes that are associated with increasing exercise reinforcement. Perhaps high-intensity interval training that induces repeated short bouts of temporary discomfort may be very effective for promoting tolerance for exercise discomfort and incentive sensitization of exercise reinforcement. Interestingly, the MET intensity of self-selected exercise training did not correlate with changes in RRV_exercise_ or Pmax_exercise_. It appears that MET intensity serves to influence tolerance for exercise intensity discomfort, which in turn influences RRV_exercise_. Thus, exercise intensity may indirectly contribute to the incentive sensitization process. These findings may have important implications for future exercise recommendations. Instead of recommending a large constant-load dose of exercise which may be inappropriate for some individuals, focusing on shorter and more intense exercise may lead to improvements in preference and tolerance and increase RRV_exercise_.

Neither exercise reinforcement (Pmax_exercise_) nor MVPA changed in any group. However, both Pmax_sed_ and time in sedentary behavior decreased in the 300 kcal group. In this respect, it is encouraging that changes in reinforcing value of a behavior aligned with changes in the usual amount of participation in that behavior. To the best of our knowledge, this is the first demonstration that the direction of change in exercise or sedentary behavior reinforcement is aligned with the direction of change in usual participation of those behaviors. Thus, the present study supports and extends previous cross-sectional research [33, 35] by demonstrating longitudinal relationships that provide stronger scientific evidence of the link between behavioral reinforcement and usual amount of behavior. The lack of change in MVPA in the 300 kcal group despite the decrease in sedentary time was due to the significant increase in time spent in light PA. Perhaps the participants in the 300 kcal group were beginning to become more active, just not at MVPA intensity.

The current study prescribed exercise sessions based on energy expenditure rather than on particular durations and intensities. Prescribing on duration was a design option, but in this case, the overall work or volume of exercise exposure would have differed between participants and likely between sessions within participants. A 30-minute walk for one participant is not equal in total work across all participants and would not induce the same total work or volume of exercise as a 30-minute run. While both the intensity and duration of the exercise could have also been controlled, due to differences in body mass of the participants, either the exercise time or total work still would have varied. Providing some freedom over the exercise parameters may promote intrinsic motivation for exercise [55, 29]. It is uncertain; however, if the results would have been different if the exercise prescription was based on overall duration of the sessions instead of kcal expended. However, the apparent importance in MET intensity for fostering greater preference + tolerance suggests that prescribing sessions based on duration of the exercise sessions would ignore the important considerations for intensity discussed above. Additional limitations include the unequal distribution of females and males (overall 83% female). The relatively small number of males did not allow for adequate comparisons of results between genders. Future investigations should include samples sizes of males and females that would allow for the disaggregation of data to test potential sex differences in incentive sensitization for exercise. Similarly, it is difficult to generalize across racial and ethnic groups as 88% of the current study’s sample was Caucasian. Exercise intensity was not controlled to provide the subjects autonomy over their exercise sessions. Perhaps exercise sessions completed at greater intensities that induced more discomfort than what is self-chosen by novice exercisers would promote greater incentive sensitization of exercise reinforcement.

## Conclusions and future directions

The exercise parameters in the present study were not prescribed to promote health and fitness, but rather to investigate whether a low-dose of exercise could promote incentive sensitization for exercise reinforcement. It may be beneficial for novice exercisers to begin exercise programs that focus on increasing exercise reinforcement and exercise as a habit before focusing on fitness and health. The low doses of exercise used in the present study were enough to decrease sedentary behavior reinforcement and shift choice away from sedentary behaviors, which helped to maintain RRV_exercise_ during and after the exercise treatment. The current longitudinal results provide extend cross-sectional evidence that increasing the preference and tolerance to exercise intensity may be important for increasing RRV_exercise_, and exercise intensity is a key component in increasing preference and tolerance for exercise intensity. Perhaps the low dose of exercise in the current study would have been able to induce incentive sensitization if the intensity of the exercise completed at each session was very great, such as occurs with high-intensity interval training.

By developing exercise programs that focus first on establishing exercise as a reinforcing behavior, individuals not engaging in exercise will be more likely to maintain an exercise program, eventually increasing their exercise capacity, and realizing the associated health benefits. The result would be more Americans meeting physical activity guidelines and enjoying better health.

## Supporting information

S1 Table CONSORT checklist. CONSORT 2010 checklist of information to include when reporting a randomized trial.
(DOC)

S1 File Research protocol. The ‘Motivation to Exercise’ study.
(DOCX)

S2 File Informed consent. Informed consent.
(PDF)
