# Supplementary material for: Inducing incentive sensitization of exercise reinforcement among adults who do not regularly exercise—A randomized controlled trial
Source: PLoS One. 2019 May 7;14(5):e0216355. doi: 10.1371/journal.pone.0216355 (PMC6504040; doi:10.1371/journal.pone.0216355)
Supplement: S1 File — The ‘Motivation to Exercise’ study. (DOCX) [file pone.0216355.s002.docx]

**The ‘Motivation to Exercise’ study**

**Principal Investigators:** James Roemmich, Ph.D, Kyle Flack, PhD

1. **Background and Significance/Relevance**

The Dietary Guidelines for Americans (DGA) provide science-based advice to promote health and to reduce risk for chronic diseases through diet and physical activity [[1](#_ENREF_1)]. However, advice alone is not effective for motivating behavior change; additional strategies are needed as only 1 in four Americans report engaging in any leisure time physical activity [[2](#_ENREF_2)], and only 9% meet the Physical Activity Guidelines for Americans [[3](#_ENREF_3)]. The low adherence of most Americans to physical activity recommendations underscores the need for research on factors affecting the adoption of two key aspects of the DGA: increased physical activity, and attainment of a healthy weight. This project addresses such factors and will yield empirical evidence that will inform physical activity guidelines and programs, as well as tools to help individuals make healthy choices regarding physical activity and maintenance of healthy body weight.

Behavioral Choice Theory provides a framework for understanding the choices people make and how to shift an individual’s choice toward healthier alternatives. The theory holds that choice is based on the relative motivating values of alternatives. One way to shift choice toward a healthier alternative is to increase the relative reinforcing value (RRV)/motivating value of that alternative. Thus, increasing the RRV of PA behaviors could shift choice towards PA and away from less healthy, sedentary alternatives. Physical activity has reinforcing properties in that exercise dependency has been demonstrated in both humans [[4-7](#_ENREF_4)] and rodents [[7-10](#_ENREF_7)]. Moreover, wheel-running in rodents produces a reinforcing aftereffect, which can be measured by conditioned place preference after the activity has ceased [[10](#_ENREF_10)]. Although human data are limited, individual differences in the RRV of physical activity (RRV_PA_) [[11-15](#_ENREF_11)] have been reported, which predict total usual PA [[16](#_ENREF_16)]. We have shown that RRV_PA_ is associated with greater health-promoting, moderate-to-vigorous intensity PA [[11](#_ENREF_11)].

Increasing the RRV_PA_ may allow PA to compete with more reinforcing, sedentary behaviors, resulting in a shift in behavioral choice. This process is termed “incentive sensitization”, which was originally proposed to explain drug addiction [[17](#_ENREF_17)]. Incentive Sensitization theory posits that the RRV of a behavior is increased through repeated exposures, which produce neuroadaptations that increase craving of the behavior [[18](#_ENREF_18)]. After repeated exposures to a stimulus, a ‘sensitization’ or hypersensitivity to the incentive motivational effects of the stimulus follows. This produces a bias of attentional processing towards the stimuli (i.e., increases its salience within the environment; incentive salience) [[19](#_ENREF_19), [20](#_ENREF_20)]. The result is an increased RRV of the stimulus relative to a competing alternative. The extent to which the RRV_PA_ can be increased has not been studied. Understanding the conditions that produce incentive sensitization for PA and the individual difference factors that may moderate the incentive sensitization process would yield a wealth of new information that could be used to help individuals make healthy choices and increase adherence to the DGA.

Another factor that influences motivation for a behavior is the development of tolerance to unpleasant aspects of that behavior. Opponent-Process Theory would account for the acquisition of motives such as use of drugs of abuse where the initial reinforcer is positive, and PA where the initial reinforcer may be negative [[21](#_ENREF_21)]. Solomon [[21](#_ENREF_21)] showed that the former case increases the affective state (State A, the rush) up to the point at which the effect of the drug wanes, resulting in an aversive-affective state characterized by craving (State B, withdrawal). Over time, State B also decays and there is a return to baseline. However, after frequent self-administrations, tolerance is developed and the positive reinforcing state (A) is greatly diminished in both intensity and duration, while the acquired motivational state (B) is heightened in both intensity and duration. Thus, both the onset of State A and the removal of the aversive State B serve to motivate behavior [[21](#_ENREF_21)].

The opposite is proposed when the initial reinforcer is negative, as may be the case for exercise. For initial exposures, the thought of exercise can produces anxiety followed by discomfort and sweating during the exercise session (State A). This is followed by relief (State B) before the ultimate return to the resting state. It is possible, however, that after many repeated exposures, tolerance may develop to the aversive aspects such that anxiety is replaced with eagerness and there is excitement and pleasure during exercise, leading to the after-reaction in State B of exhilaration followed by a gradual return to a resting state. Opponent Process Theory predicts that the affect experienced after repeated exposures is opposite to that experienced during the first few presentations [[21](#_ENREF_21)]. For exercise, this would include tolerance to any discomfort, pain, fatigue and displeasure and a greater positive post-exercise affective response [[22](#_ENREF_22)]. This is important, as the affect experienced during and after exercise is known to have a greater motivational significance in PA participation than knowledge and beliefs regarding health benefits[[22](#_ENREF_22)]. Therefore, we propose that repeated exposures to PA will produce tolerance to any unpleasant aspects while increasing the post-exercise affect [[23](#_ENREF_23), [24](#_ENREF_24)].

Sensitizing PA reinforcement and increasing discomfort tolerance may be associated with the duration and intensity of daily activity bouts [[6](#_ENREF_6), [25-28](#_ENREF_25)]. We anticipate that a moderate PA dose (300 kcal expenditure) will produce greater incentive sensitization and tolerance than a low dose (150 kcal). This is based on observations that the development of sensitization of drugs of abuse is dose-dependent, with mid-range doses increasing reinforcement more than either low or very high doses [[29](#_ENREF_29)].

This study will address the primary question of:

- Can repeated exposure to PA increase the RRV_PA_ in sedentary individuals?

This study will address the secondary/exploratory questions of:

- Can repeated exposure to PA increase tolerance to aversive aspects of PA?
- Is increased RRV_PA_ associated with increased usual PA?
- Is increased discomfort tolerance associated with increased usual PA?

1. **Hypotheses to be Tested**

**Primary hypothesis**

*Hypothesis 1:* 300 kcal doses of PA will increase RRV_PA_ more than 150 kcal doses and no PA.

**Secondary/Exploratory hypotheses**

*Hypothesis 2:* 300 kcal doses of PA will produce greater exercise discomfort tolerance than 150 kcal doses and no PA.

*Hypothesis 3:* Increases in the RRV_PA_ will be positively associated with the change in usual PA.

*Hypothesis 4:* Increases in discomfort tolerance will be positively associated with the change in usual PA.

| Table 1. Design of experiment | | | | | |
| --- | --- | --- | --- | --- | --- |
| Sex | PA dose | 0 wk | 6 wk | | 10 wk |
|  |  | <-treatment-> | |  |  |
| Male | 0 kcal (n=15) | X | X | | X |
|  | 150 kcal (n=15) | X | X | | X |
|  | 300 kcal (n=15) | X | X | | X |
|  |  |  |  | |  |
| Female | 0 kcal (n=15) | X | X | | X |
|  | 150 kcal (n=15) | X | X | | X |
|  | 300 kcal (n=15) | X | X | | X |

**C. Methods**

**i. Participants and screening**: As shown in Table 1, 45 men and 45 women age 18-49 y, that range in adiposity from lean to obese (19-35 kg/m^2^) and have no contraindications for PA participation will be recruited. Subjects will be sedentary, i.e. not be regularly engaging in exercise more than once per week. They will be instructed to not enroll in an exercise program and to limit planned PA to that prescribed by the investigators for the duration of the incentive sensitization treatment. Subjects cannot have high occupational activity such as construction trades, farm workers, and freight movers who complete most of their work by hand. Additional exclusionary criteria include a liking of <3 out of 10 for all available PA modes and exercising for more than 30 min/day > 2d/wk and repeated non-compliance with completing the exercise sessions. Participants will be screened for entry criteria and will report the times/wk they engage in various types of PA.

**ii. Design:** Physical activity will be performed on stationary cardio-type exercise machines (choice each day of treadmill, elliptical, stair stepper, cycle). Each treatment will be 6 wk in duration with 3 treatment sessions/wk. Outcome testing will occur at baseline, 3, 6, 10, and 14 wks. As shown in **Table**

**1**, participants will be randomized to exposures of 0, 150, or 300 kcal/session. The 150 kcal and 300 kcal dose groups will participate in 3 physical activity sessions (days)/wk. All participants will be provided Choice Health and Fitness (CHF) memberships, the 0 kcal dose group will receive their CHF membership and exercise program after they complete the study.

**iii. Procedures**:

Visit One: Screening and Consent

Participants will be recruited by Grand Forks Human Nutrition Research Center (GFHNRC) staff using Survey Monkey. We will leverage our partnership with Choice Health and Fitness to utilize their electronic entry records and send recruitment materials to those fitness club members who attend Choice Fitness 2 or fewer visits per week and otherwise seem to meet our other entry criteria. Various other media will be used to advertise the study. This may include brochures, print media such as the Grand Forks Herald, Dakota Student, Exponent, and Tidbits, television and radio such as the UND television and radio stations. Internet advertisements including University Research Newsletter, University Letter, HEALTHY-UND Listserv, and Work Well Newsletter may be utilized. Recruitment efforts may also include posting flyers around the Greater Grand Forks area and surrounding communities, promoting the study at expos and trade shows, and providing information on the GFHNRC website. Recruitment will continue until the study needs are met. A sample advertisement is attached (APPENDIX 1 a/b). Subjects may apply to join the study by completing the on-line application on Survey Monkey (APPENDIX 2). Eligible applicants will be invited to an information/screening appointment at the GFHNRC or CHF. During this initial visit, subjects will be informed about the study and have any questions answered. If deemed eligible and the subject desire to participate, they will be asked to provide written informed consent (APPENDIX 3). After signing the consent, subjects will fill out a demographic form (APPENDIX 4), a medical history form (APPENDIX 5), and be measured for anthropometrics. Subjects will also be provided a SenseWear device to wear for the following week to monitor free-living energy expenditure and physical activity. Questionnaires including The Preference for and Tolerance of the Intensity of Exercise Questionnaire (PRETIE-Q) (Appendix 6) [[30](#_ENREF_30), [31](#_ENREF_31)], the Discomfort Intolerance Scale [[32](#_ENREF_32)] (Appendix 7), Social norms [[33](#_ENREF_33)], Social Support and Exercise Survey (APPENDIX 8) [[34](#_ENREF_34)], the Exercise Self-Efficacy Scale (EXSE, Appendix 9) [[35](#_ENREF_35)] and the Exercise Confidence Survey (APPENDIX 10), Psychological needs satisfaction (PNSE) (APPENDIX 11), and the Behavioral Regulation in Exercise questionnaire (BREQ-3) (APPENDIX 12) will be completed by the subject at this time. The first of four dietary recalls will be completed and the subject will be scheduled for their three baseline visits at (CHF).

Baseline Assessment Day 1 (CHF)

Subjects will arrive at CHF at least 2 hrs after eating to complete the physical activity reinforcement task (RRV_PA_). Participants will first rate their liking of the PA and sedentary alternatives (Appendix 13). The highest rated PA and sedentary alternatives will be provided as alternatives for the RRV testing session. Additionally, treatment expectations of the study will be assessed with the Stanford Expectations of Treatment Scale (SETS) [[36](#_ENREF_36)] tailored for the current study (APPENDIX 14). The SETS tool assesses expectancy effects to be co-varied for in the statistical analyses. Participants will complete a dietary recall.

Baseline Assessment Day 2 (CHF)

Subjects will arrive at CHF at least 2 hrs after eating to complete body composition and aerobic fitness tests. Body composition will be determined by DXA and submaximal fitness tests will be completed on a motorized treadmill. Participants will complete a dietary recall.

Baseline Assessment Day 3 (CHF)

Subjects will arrive at CHF at least 2 hrs after eating to complete an affect-during-exercise treadmill test. Affect will be measured using single item scales including the Feeling scale (APPENDIX 15) to measure affective valence [[37](#_ENREF_37)], Felt Arousal Scale (APPENDIX 16) to measure perceived activation [[38](#_ENREF_38)], and Borg’s Rating of Perceived Exertion scale (APPENDIX 17) to measure perceived effort [[39](#_ENREF_39)]. Participants will also complete muscular pain scales (APPENDIX 18) during exercise and the multi-item Activation-Deactivation Adjective Check-list (APPENDIX 19) pre-post exercise as a measure of energetic arousal and tense arousal [[40](#_ENREF_40)]. Affect, perceived exertion, arousal, and muscular pain will be measured using single item scales at rest, the last 30 s of a 3 min warm-up (40% < ventilatory threshold (VT)), the last 30 s of every 3 min during 12 min of exercise at an intensity 20% < VT, the last 30 s of every 3 min during 12 min of exercise at an intensity 10-15% > VT, at the end of a 5 min cool down (at an exercise intensity of 20% < VT) and after 10 minutes of rest. Participants will complete the multi-item Activation Deactivation Adjective Check List (APPENDIX 17) before and after completing the exercise. During rest and exercise, heart rate will be measured via telemetry from a chest band (Polar Electro Inc., Lake Success, NY). Beat-to-beat blood pressure, stroke volume, total peripheral resistance, cardiac output, and pulse rate variability will be monitored using a Finometer PRO (Finapres Medical Systems, Amsterdam). Participants will complete a dietary recall and be scheduled for initiation of the 6-week intervention period.

6-Week Intervention The treatment is based on meeting the prescribed kcal expenditure as measured using the SenseWear device (described below) three times per week. The intensity, duration, and interval/ constant load nature of physical activity will not be prescribed. For approximately the first six sessions, a research assistant will be present to provide instruction on the use of the stationary exercise machines and will monitor each session for attaining the energy expenditure goal of either 150 kcal or 300 kcal. The study-related exercise will be a circumscribed and intentional bout recorded by the participant in a log book. Compliance regarding the energy expenditure of each physical activity session will be monitored by the subject and staff using an activity monitor. A research assistant will meet with the subject each week at the fitness center to download and review the activity monitor wear log (APPENDIX 20) and activity monitor data with the volunteer. Volunteers will be given feedback if the exercise bout cannot be readily identified from the activity monitor or if the bout did not meet the energy expenditure criteria. To promote external validity and minimize seasonal/weather effects, treatment sessions will occur at the fitness center. Subjects in the 0 kcal group will wear the physical activity monitor only during assessment periods. No instruction will be provided regarding dietary intake as spontaneous change in dietary intake is an outcome measure.

Week-6 Assessments (Post)

At the conclusion of the intervention, participants will follow the testing schedule outlined for the baseline assessments. Additionally, the Perceived Autonomy support questionnaire will be administered (APPENDIX 21) and subjects will be issued an activity monitor to wear for the following week and will return to CHF to return the device.

Week 10 Assessments

After four weeks of no-contact with the study investigators, subjects will follow the testing schedule outlined for the baseline assessments except they will not complete a body composition test. Subjects will be issued an activity monitor to wear for the following week and will return to CHF to return the device.

**iv. Measures**: Gender, baseline age, and baseline values of outcomes will be tested as moderators of change in RRV of physical activity (**Table 2**).

**Table 2- Measures and testing schedule**

| Measure |  | 0 wk | 6 wk | 10 wk |
| --- | --- | --- | --- | --- |
| RRV & liking of activity | 1^O^ outcome | X | X | X |
| Usual physical activity | 2^O^ outcome | X | X | X |
| Body composition | 2^O^ outcome | X | X |  |
| Aerobic fitness | 2^O^ outcome | X | X | X |
| Muscular pain | 2^O^ outcome | X | X | X |
| Dietary intake | 2^O^ outcome | X | X | X |
| Questionnaires |  |  |  |  |
| Self-efficacy | 2^O^ outcome | X | X | X |
| Affect during exercise | 2^O^ outcome | X | X | X |
| Social norm of exercise | 2^O^ outcome | X | X | X |
| Tolerance of discomfort | 2^O^ outcome | X | X | X |
| Psychological needs satisfaction | 2^O^ outcome | X | X | X |
| Behavioral regulation in exercise | 2^O^ outcome | X | X | X |
| Perceived autonomy support | 2^O^ outcome |  | X |  |

**Physical activity reinforcement task and liking of physical activity:** The RRV_PA_ assessed by evaluating the number of responses (mouse button presses) a participant is willing to complete to gain access to a PA or alternative sedentary reinforce [[41](#_ENREF_41), [42](#_ENREF_42)]. Just as a rat will lever press to receive a chow pellet, people will press a button to get access to behavioral alternatives. For a rat, the number of presses required to obtain the food reinforcer can be increased over time to determine the reinforcing value of food. Food is more reinforcing for rats that are willing to press a greater number of times to receive the food. For humans, the procedure begins by determining the liking of the physical activity and sedentary alternatives using a 10-point Likert-type scale (1 = “do not like at all” and 10 = “like very much”) and choosing the highest rated PA for the testing session. A sedentary alternative is included so that the subjects are not responding for exercise out of boredom (there is nothing else to do). The lab space includes two workstations. One station is a computer and mouse on which the participant can earn points towards their highest liked physical activity (e.g., treadmill, cycle ergometer, elliptical step ergometer). At the other station is a computer to earn points towards the highest liked sedentary alternative (reading magazines, paper-pencil puzzles). Participants can switch between stations as much as they choose. The program presents a game that mimics a slot machine. A point is earned each time that the shapes match. For every 3 points, the subject will receive 2 min of access to reinforcer that was earned. At first, points are delivered after every 4 presses, but then doubles (8, 16, 32, […] 1024) each time 3 points are earned. For instance, the participant initially has to click the mouse 4 times to earn each point, but after the first 3 points and reward is earned, then 8 clicks are required to earn each of the 3 points, and so on [[41](#_ENREF_41), [42](#_ENREF_42)]. Participants will engage in each alternative reinforcer for the time earned at the end of the session.

**Physical activity***.* Physical activity duration and energy expenditure during each treatment session and during free-living physical activity will be measured using a SenseWear Mini® [[43](#_ENREF_43)] or Computer Science and Applciations (CSA) monitor.

**Body composition.** Height and weight will be measured and BMI calculated. Body composition will be assessed with total body DXA (iDXA, GE Lunar).

**Aerobic fitness***.* Subjects will complete submaximal fitness tests on a motorized treadmill. Given the sedentary nature of the participants, the test will be stopped when the subject reports a rating of perceived exertion (RPE) of > 7, achieves a HR associated with 85% of predicted % heart rate reserve (%HRR), or 3) a respiratory exchange ratio of 1.0. These parameters allow sedentary subjects to exceed VT by 2 or more stages so that VT can be determined [[44](#_ENREF_44)]. Heart rate measured via telemetry from a chest band (Polar Electro Inc., Lake Success, NY) and metabolic data via indirect calorimetry (TrueOne 2400, ParvoMedics, Sandy UT) will be continuously monitored. VT will be determined from plots of the ventilatory equivalent for oxygen (V_E_/VO_2_) and the ventilatory equivalent for carbon dioxide (V_E_/VCO_2_) versus time. VT is identified as the workload before V_E_/VO_2_ begins to increase without an increase in the V_E_/VCO_2_. The HR and workload corresponding to VT will be determined for the test of affect during exercise and for physical activity prescription. Beat-to-beat blood pressure stroke volume, total peripheral resistance, cardiac output, and pulse rate variability will be monitored continuously during the exercise test using a finger cuff and infrared plethysmograph (Finometer PRO, Finapres Medical Systems, Amsterdam).

**Feeling Scale (FS)**. The FS (APPENDIX 15) [[45](#_ENREF_45)] is a single-item measure of affective valence and ranges from -5 to +5 with descriptive anchors at 0 (neutral) and all odd integers, anchored by “Very Bad” [[46](#_ENREF_46)] to “Very Good” (+5) [[45](#_ENREF_45)]. The Feeling Scale has a moderate to strong correlation (0.51 to 0.88) with the Valence scale of the Self-Assessment Manikin [[47](#_ENREF_47)] and from 0.41 to 0.59 with the Valence scale of the Affect Grid [[48](#_ENREF_48)].

**Felt Arousal Scale (FAS)**. The FAS (APPENDIX 16) [[38](#_ENREF_38)] is a six-point, single-item measure of perceived activation that ranges from 1 to 6 with anchors of “Low Arousal” and “High Arousal” [[38](#_ENREF_38)]. The FAS correlates with the Arousal scale of the SAM ranging from 0.45 to 0.70 and with the Arousal scale of the AG from 0.47 to 0.65 [[47](#_ENREF_47)].

**Perceived Exertion**.

Borg’s Rating of Perceived Exertion (RPE) scale (APPENDIX 17) will be used to measure perceived effort [[39](#_ENREF_39)] during the affect-during-exercise treadmill test at the same time points as the FS and FAS (above) as a measure of perceived exercise intensity. The weighted mean validity coefficients for Borg’s RPE scale are 0.62 for heart rate, 0.57 for blood lactate, 0.64 for %VO2max 0.63 for VO2, 0.61 for ventilation and 0.72 for respiration rate [[49](#_ENREF_49)].

**Muscular pain***.* Pain intensity of the whole body, shoulders and legs will be measured at rest, each stage of the fitness test, during cool down, and 10 min after the test using the muscular pain scale modeled after Borg’s category-10 ratio scale (APPENDIX 18) [[50](#_ENREF_50)]. Muscular pain and exertion will be measured at the same time points as the FAS, FS, and RPE scale during the affect-during-exercise treadmill test [[51](#_ENREF_51)].

Figure 1. Circumplex model

**Activation Deactivation Adjective Check List (ADCL)**


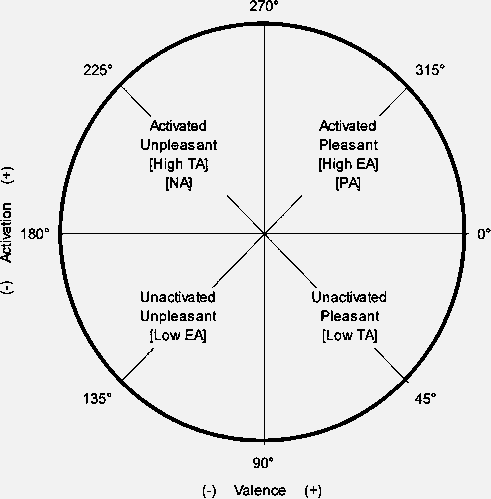
The AD ACL [[40](#_ENREF_40)] is a multi-item scale that measures the affective dimensions of Energetic Arousal (EA) and Tense Arousal (TA) and will be administered pre and post exercise. The AD ACL (APPENDIX 19) consists of 20 self-descriptive adjectives of Energy, Tiredness, Tension, and Calmness. The AD ACL is scored by assigning 4, 3, 2, and 1, respectively to the "√√,√, ?" and "no" scale points, and summing the five scores for each adjective. In order of appearance, the adjectives are as follows: Energetic (active, energetic, vigorous, lively, full-of-pep); Tired (sleepy, tired, drowsy, wide-awake, wakeful); Tension (jittery, intense, fearful, clutched-up, tense); Calmness (placid, calm, at-rest, still, quiet). Scoring for "wakeful" and "wide-awake" must be reversed for the Tiredness adjective. Tiredness and Tension scores must be reversed (but not wakeful and wide-awake in this case) before summing the ten scores. The FS, FAS, and AD ACL will be displayed from the perspective of the circumplex model. As shown in **Figure 1**, the energy pole (high EA) maps the high-activation pleasure quadrant. Tension (high TA) maps the high activation displeasure quadrant. Tiredness (low EA) maps the low-activation displeasure quadrant, and calmness (low TA) maps the low-activation pleasure quadrant [[40](#_ENREF_40), [52](#_ENREF_52)]. Subjects will complete the AD ACL immediately before, after, and 10 minutes after the affect-during-exercise treadmill test. Values from the FS, FAS, and AD ACL will be calculated seperatly at each time point and plotted on the circumplex model to display changes in affect throughout the exercise bout and pre-post exercise [[53](#_ENREF_53)].

**Tolerance for the discomfort of exercise***.* The Preference for and Tolerance of the Intensity of Exercise Questionnaire (APPENDIX 6) [[30](#_ENREF_30), [31](#_ENREF_31)], and the Discomfort Intolerance Scale [[32](#_ENREF_32)] (APPENDIX 7) will be completed. These measures will be tested as predictors of RRV_exercise_ as both preference and tolerance are related to the frequency of participation in strenuous exercise and the total leisure-time exercise [[30](#_ENREF_30)].

**Self-efficacy for exercise***.* Self-Efficacy will be assessed using the Exercise Self-Efficacy Scale (EXSE, Appendix 4) [[35](#_ENREF_35)] and the Exercise Confidence Survey (Appendix 9) [[54](#_ENREF_54)] The EXSE assesses an individual’s beliefs in their ability to continue to exercise at moderate intensity for 40 or more minutes per session, 3 times/wk.  Participants score each item on a 100-point scale ranging from 0% (not at all confident) to 100% (highly confident).  For the Exercise Confidence Survey participants rate how confident they are that they could really motivate her/himself to do things related to trying to increase or continue regular exercise, for at least six months. Participants score each item on a 5-point scale ranging from 1 (I know I cannot) to 5 (I know I can).

**Social norms and support for exercise***.* Social norms will be measured using subjective and descriptive norm items (APPENDIX 8) [[33](#_ENREF_33)]. Social support will be assessed using the Social Support and Exercise Survey [[34](#_ENREF_34)] which tests both family and friend participation in exercise with the subject. Participants first rate how often anyone in their family and then how often their friends have said or done various aspects related to social norms and support of exercise. Participants score each item on a scale ranging from 1 (none) to 5 (very often).

**Psychological Need Satisfaction in Exercise (PNSE).** The PNSE is an 18-item self-report questionnaire that assesses respondents’ perceptions of competence, autonomy, and relatedness felt during exercise (APPENDIX 11) [[55](#_ENREF_55)]. Each item is scored on a 6-point Likert scale (1 = not at all true… 6 = very true) in terms of how they feel while exercising. The PNSE has excellent structural and convergent validity and internal consistency of 0.90 to 0.91 for each of the competence, autonomy, and relatedness subscales in physically active young adults [[55](#_ENREF_55)].

**Behavioral Regulation in Exercise Questionnaire (BREQ).** The BREQ is a 15-item self-report measure that assesses external, identified, intrinsic, and introjected regulations (APPENDIX 12) [[56](#_ENREF_56)]. Each item is scored on a 5-point scale ranging from 0 (not true for me) to 4 (very true for me). Validity of the BREQ’s four-factor structure, the gender invariance of that factor structure, and the internal consistency (0.76 to 0.90) of the subscales has been established [[56](#_ENREF_56)].

**Sport Climate Questionnaire.** Perceived autonomy support from the coach/trainer is measured using the short (6 items) version of the Sport Climate Questionnaire (APPENDIX 21). The original scale [[57](#_ENREF_57)] that assesses perceptions of autonomy support provided by a health care provider was revised by the original authors such that the term ‘‘my health care provider’’ was replaced with ‘‘my exercise class leader’’. Moreover, participants respond to the items in reference to the first 2 weeks of exercise sessions monitored by GFHNRC staff. Each item is scored on a 7-point scale ranging from 1 (strongly disagree) to 7 (strongly agree). The original questionnaire [[57](#_ENREF_57)] has a one-factor solution that measures perceived autonomy support and internal consistency of 0.95.

**Dietary intake***.* The Automated Self-Administered 24-Hour Dietary Recall (ASA24) will be used to collect dietary information. Subjects will report dietary information for 3 weekdays and 1 weekend day each testing period. The ASA24 is based on the multi-pass method [[58](#_ENREF_58)] and has been shown to effectively assess mean energy intake within 10% actual intake [[59](#_ENREF_59)].

**Expectations***.* Subject outcome responses to a treatment may be influenced by the expectations that they have before starting treatment. Treatment expectations of the study will be assessed with the Stanford Expectations of Treatment Scale (SETS) tailored for the current study (APPENDIX 14). The SETS tool assesses expectancy effects that can be covaried for in the statistical analyses. Treatment expectations will be assessed at baseline.

**Goals***.* After the subjects have completed the 6 weeks of treatment, they will be asked to detail any new exercise or sports participation goals they have made since beginning study participation.

**D. Statistical Analysis Plan**

Differences in groups across time in the primary outcome (RRV_PA_) will be tested using a General Linear Model with treatment group and sex treated as between factors and time (baseline, 6, 10 wk) as a within factor, baseline RRV_PA_ as a covariate and % body fat as a moderator variable. ***Power analysis***: based on demonstrating initial increases in RRV_PA_ of physical activity during the 6 wk treatment period. Assuming α=0.05 and a between-subject SD=8, 15 subjects/group are needed to have 90% power to detect a mean difference in RRV_PA_ of 7 (40% difference) between the 2 levels of physical activity treatment groups at the end of the 6 wk treatment period. Previous studies [[16](#_ENREF_16), [18](#_ENREF_18)] have reported a between subject SD for the RRV_PA_ to range from 35% to 55% of the mean. An SD of 8 represents the midpoint of this range. Therefore, goal recruitment will be 15 subjects per study group.

**E. Anticipated Results and Likely Impact**

We expect that a moderate dose of exercise, a lower intensity of PA exposures, and an interval pattern of exposures will produce greater increases in physical activity reinforcement. The dose prediction is based on findings from the drugs of abuse literature where mid-range doses increase reinforcement more than low or very high doses [[29](#_ENREF_29)]. The objectives of this proposal focus on controlled individual-level treatments to promote healthy choices. Results from this project will inform future community-based behavioral interventions that will provide evidence for guidelines, treatments, and/or tools that can be used by health care providers, policy makers, and stakeholders to help people meet the DGA.

**F. Human Subject Burden (Time, Discomfort, Inconvenience)**

**Table 4.** Time to complete each measurement

|  | # Days assessed | Min to complete/day |
| --- | --- | --- |
| RRV and liking of activity | 3 | 60 |
| Habitual PA | 21 (3 weeks) |  |
| Body composition (DXA) | 2 | 15 |
| Aerobic fitness | 3 | 30 |
| Muscular pain | 3 | 10 |
| Energy/nutrient intake | 12 | 15 |
| Affect | 3 | 30 |
| Exercise questionnaires(6) | 3 | 10 |
| Perceived autonomy support | 1 | 10 |

**G. Requested Subject Reimbursement Schedule(s)**

Reimbursement for the total study is $500/subject or a Choice Health & Fitness membership equivalent to $600/subject.

This will be prorated based on completion of baseline ($150), end of training ($50), and end of follow-up ($300) measures.

**H. Impact on Center Resources**

**i. Budget**

Source of Funds: Center Funds. See budget in **Table 5**.

**Table 5**. *Total project budget*

| item | | | cost, $ | items/subject | Cost/subject, $ | total cost to project, $ (90 subjects) |
| --- | --- | --- | --- | --- | --- | --- |
| Recruitment/ screening |  | | 2000 |  |  | 2,000 |
| Assessments |  | |  |  |  |  |
|  | PA | | 0 | 3 | 0 | 0 |
|  | RRV_PA_ | | 0 | 3 | 0 | 0 |
|  | Questionnaires | | 0 | 19 | 0 | 0 |
|  | DXA | | 10 | 2 | 0 | 0 |
|  | Aerobic fitness | | 10 | 3 | 5 | 450 |
|  | Energy/nutrient intake | | 0 | 12 | 0 | 0 |
|  | Affect test | | 0 | 3 | 0 | 0 |
|  |  | |  |  |  |  |
| Subject stipends |  | | 500 | 1 | 500 | 45,000 |
| Choice Memberships (6 months) | |  | 318 | 1 | 318 | 28,620 |
| Grand Total |  | |  |  |  | 74,070 |

ii.. **Support Staff Time Needed**

*Applied Physiology Laboratory, Nurse Coordinator, and allied staff*:

Recruiting: 90-180 hrs. (1-2 hours per participant)

Screening and consenting: 90 hrs. It is estimated that 1 hrs/participant is needed to screen and consent each

CAReS scheduling: 180 hrs. 2 hours per participant for set-up and maintenance of CAReS throughout the study.

Exercise monitoring: 1440 hrs. It is estimated that on average 24 hr of monitoring will be needed per subject. For 60 participants in exercise groups, 1440 hrs are needed.

Anthropometry (height, weight) - 225 hrs (1 h of direct participant time, 0.5 hr prep and clean-up, 1 hr for data reduction and transfer). For 90 participants, 225 hrs are needed.

Questionnaires – 23.75 hrs (0.25 hr for direct participant time, 1 hr for data reduction and transfer). For 90 participants completing 6 different questionnaires measured on 3 occasions, and one questionnaire measured on one occasion, 23.75 hrs total are needed.

RRV of activity – 607.5 hrs (1.0 h of direct participant time, 0.75 hr preparation and clean-up, 0.5 hr for data reduction and transfer). For 90 participants measured on 3 occasions, 607.5 hrs total are needed.

DXA - 162 hrs (0.3 h for direct participant time, 0.5 hr preparation and clean-up, 1 hr for data reduction and transfer). For 90 participants measured on 3 occasions, 162 hrs total are needed.

Aerobic fitness- 742.5 hrs (1.0 h for direct participant time, 0.75 hr prep and clean-up, 1 hr for data reduction and transfer). For 90 participants measured on 3 occasions, 742.5 hrs total are needed.

*Dietitians:*

Energy intake (food recalls) - 90 hrs; 1.0 hr for reminder calls and checking adequacy of online recalls per participant.

*Statistics:* 24 hrs.

*Information Technology:* 12 hrs. The IT staff estimates 12 hr is needed for CAReS and other data handling.

iii. **Facilities Needed**: This study will utilize mainly the Choice Health and Fitness Laboratory for consenting and volunteer coordination, DXA, fitness measures, phlebotomy, and measures of motivation, and exercise training. The Metabolic Kitchens will be used for meal preparation. Statistical consultation will be provided by LuAnn Johnson.

**I. Subject Recruitment Plan.** Participants will be recruited by the Grand Forks Human Nutrition Research Center (GFHNRC) staff using Survey Monkey. Various media will be used to advertise the study. This may include brochures, print media such as the Grand Forks Herald, Dakota Student, Exponent, and Tidbits, television and radio such as the UND television and radio stations. Internet advertisements including University Research Newsletter, University Letter, HEALTHY-UND Listserv, and Work Well Newsletter may be utilized. Recruitment efforts may also include posting flyers around the Greater Grand Forks area, promoting the study at expos and trade shows, and providing information on the GFHNRC website. Recruitment will continue until the study needs are met.

**J. Timetable**. It is anticipated that submission to the Institutional Review Board will be completed by January 13, 2015 for approval on February 6, 2015.

**References:**

1. *Physical activity guidelines for Americans.* Okla Nurse, 2008. **53**(4): p. 25.

2. Moore, L.V., et al., *Trends in no leisure-time physical activity--United States, 1988-2010.* Res Q Exerc Sport, 2012. **83**(4): p. 587-91.

3. Tucker, J.M., G.J. Welk, and N.K. Beyler, *Physical activity in U.S.: adults compliance with the Physical Activity Guidelines for Americans.* Am J Prev Med, 2011. **40**(4): p. 454-61.

4. Chan, C.S. and H.Y. Grossman, *Psychological effects of running loss on consistent runners.* Percept Mot Skills, 1988. **66**(3): p. 875-83.

5. Chapman, C.L. and J.M. De Castro, *Running addiction: measurement and associated psychological characteristics.* J Sports Med Phys Fitness, 1990. **30**(3): p. 283-90.

6. Holden, C., *Compulsive behaviors: "Behavioral' addictions: Do they exist?* Science, 2001. **294**(5544): p. 980-982.

7. Belke, T.W., *Running and responding reinforced by the opportunity to run: Effect of reinforcer duration.* Journal of the Experimental Analysis of Behavior, 1997. **67**(3): p. 337-351.

8. Belke, T.W., *Studies of wheel-running reinforcement: parameters of Herrnstein's (1970) response-strength equation vary with schedule order.* J Exp Anal Behav, 2000. **73**(3): p. 319-31.

9. Iversen, I.H., *Techniques for Establishing Schedules with Wheel Running as Reinforcement in Rats.* Journal of the Experimental Analysis of Behavior, 1993. **60**(1): p. 219-238.

10. Lett, B.T., et al., *Pairings of a distinctive chamber with the aftereffect of wheel running produce conditioned place preference.* Appetite, 2000. **34**(1): p. 87-94.

11. Roemmich, J.N., et al., *Association of liking and reinforcing value with children's physical activity.* Physiol Behav, 2008. **93**(4-5): p. 1011-8.

12. Barkley, J.E., L.H. Epstein, and J.N. Roemmich, *Reinforcing value of interval and continuous physical activity in children.* Physiol Behav, 2009. **98**(1-2): p. 31-6.

13. Epstein, L.H., et al., *The motivation to be sedentary predicts weight change when sedentary behaviors are reduced.* International Journal of Behavioral Nutrition and Physical Activity, 2011. **8**.

14. Epstein, L.H., et al., *The value of sedentary alternatives influences child physical activity choice.* International Journal of Behavioral Medicine, 2004. **11**(4): p. 236-242.

15. Epstein, L.H., et al., *Behavioral Economic-Analysis of Activity Choice in Obese Children.* Health Psychology, 1991. **10**(5): p. 311-316.

16. Epstein, L.H., et al., *Reinforcing value of physical activity as a determinant of child activity level.* Health Psychology, 1999. **18**(6): p. 599-603.

17. Robinson, T.E. and K.C. Berridge, *The neural basis of drug craving: an incentive-sensitization theory of addiction.* Brain Res Brain Res Rev, 1993. **18**(3): p. 247-91.

18. Salvy, S.J., et al., *Effect of peers and friends on youth physical activity and motivation to be physically active.* J Pediatr Psychol, 2009. **34**(2): p. 217-25.

19. Robinson, T.E. and K.C. Berridge, *Review. The incentive sensitization theory of addiction: some current issues.* Philos Trans R Soc Lond B Biol Sci, 2008. **363**(1507): p. 3137-46.

20. Epstein, L.H. and J.N. Roemmich, *Reducing sedentary behavior: role in modifying physical activity.* Exerc Sport Sci Rev, 2001. **29**(3): p. 103-8.

21. Solomon, R.L., *The opponent-process theory of acquired motivation: the costs of pleasure and the benefits of pain.* Am Psychol, 1980. **35**(8): p. 691-712.

22. Ekkekakis, P., G. Parfitt, and S.J. Petruzzello, *The Pleasure and Displeasure People Feel When they Exercise at Different Intensities Decennial Update and Progress towards a Tripartite Rationale for Exercise Intensity Prescription.* Sports Medicine, 2011. **41**(8): p. 641-671.

23. Heishman, S.J. and J.E. Henningfield, *Tolerance to repeated nicotine administration on performance, subjective, and physiological responses in nonsmokers.* Psychopharmacology, 2000. **152**(3): p. 321-333.

24. Riley, A.L., *The paradox of drug taking: The role of the aversive effects of drugs.* Physiology & Behavior, 2011. **103**(1): p. 69-78.

25. Christie, M.J. and G.B. Chesher, *Physical-Dependence on Physiologically Released Endogenous Opiates.* Life Sciences, 1982. **30**(14): p. 1173-1177.

26. Kagan, D.M. and R.L. Squires, *Addictive Aspects of Physical Exercise.* Journal of Sports Medicine and Physical Fitness, 1985. **25**(4): p. 227-237.

27. Tate, A.K. and S.J. Petruzzello, *Varying the intensity of acute exercise: Implications for changes in affect.* Journal of Sports Medicine and Physical Fitness, 1995. **35**(4): p. 295-302.

28. Ferreira, A., et al., *Spontaneous appetence for wheel-running: a model of dependency on physical activity in rat.* European Psychiatry, 2006. **21**(8): p. 580-588.

29. Liu, Y., D.C.S. Roberts, and D. Morgan, *Sensitization of the reinforcing effects of self-administered cocaine in rats: effects of dose and intravenous injection speed.* European Journal of Neuroscience, 2005. **22**(1): p. 195-200.

30. Ekkekakis, P., et al., *The preference for and tolerance of the intensity of exercise questionnaire: psychometric evaluation among college women.* Journal of Sports Sciences, 2008. **26**(5): p. 499-510.

31. Ekkekakis, P., E.E. Hall, and S.J. Petruzzello, *Some like it vigorous: Measuring individual differences in the preference for and tolerance of exercise intensity.* Journal of Sport & Exercise Psychology, 2005. **27**(3): p. 350-374.

32. Schmidt, N.B., J.A. Richey, and K.K. Fitzpatrick, *Discomfort intolerance: Development of a construct and measure relevant to panic disorder.* J Anxiety Disord, 2006. **20**(3): p. 263-80.

33. Trinh, L., et al., *Correlates of physical activity in a population-based sample of kidney cancer survivors: an application of the theory of planned behavior.* International Journal of Behavioral Nutrition and Physical Activity, 2012. **9**.

34. Sallis, J.F., et al., *The development of scales to measure social support for diet and exercise behaviors.* Prev Med, 1987. **16**(6): p. 825-36.

35. McAuley, E., *Self-efficacy and the maintenance of exercise participation in older adults.* Journal of Behavioral Medicine, 1993. **16**(1): p. 103-13.

36. Younger, J., et al., *Development of the Stanford Expectations of Treatment Scale (SETS): a tool for measuring patient outcome expectancy in clinical trials.* Clin Trials, 2012. **9**(6): p. 767-76.

37. Hardy CJ, R.W., *Not what, but how one feels: the measurement of affect during exercise.* J Sport Exerc Psychol, 1989. **11**: p. 13.

38. Svebak, S. and S. Murgatroyd, *Metamotivational Dominance - a Multimethod Validation of Reversal Theory Constructs.* Journal of Personality and Social Psychology, 1985. **48**(1): p. 107-116.

39. Borg, G., P. Hassmen, and M. Lagerstrom, *Perceived exertion related to heart rate and blood lactate during arm and leg exercise.* Eur J Appl Physiol Occup Physiol, 1987. **56**(6): p. 679-85.

40. Thayer, R.E., *Factor analytic and reliability studies on the Activation-Deactivation Adjective Check List.* Psychol Rep, 1978. **42**(3 PT 1): p. 747-56.

41. Bickel, W.K., L.A. Marsch, and M.E. Carroll, *Deconstructing relative reinforcing efficacy and situating the measures of pharmacological reinforcement with behavioral economics: a theoretical proposal.* Psychopharmacology, 2000. **153**(1): p. 44-56.

42. Epstein, L.H., et al., *Food reinforcement, energy intake, and macronutrient choice.* American Journal of Clinical Nutrition, 2011. **94**(1): p. 12-18.

43. Johannsen, D.L., et al., *Accuracy of armband monitors for measuring daily energy expenditure in healthy adults.* Med Sci Sports Exerc, 2010. **42**(11): p. 2134-40.

44. Gaskill, S.E., et al., *Validity and reliability of combining three methods to determine ventilatory threshold.* Med Sci Sports Exerc, 2001. **33**(11): p. 1841-8.

45. Hardy, C.J. and W.J. Rejeski, *Not What, but How One Feels - the Measurement of Affect during Exercise.* Journal of Sport & Exercise Psychology, 1989. **11**(3): p. 304-317.

46. Yeh, M.C., et al., *Understanding barriers and facilitators of fruit and vegetable consumption among a diverse multi-ethnic population in the USA.* Health Promotion International, 2008. **23**(1): p. 42-51.

47. Ekkekakis, P., E.E. Hall, and S.J. Petruzzello, *The relationship between exercise intensity and affective responses demystified: To crack the 40-year-old nut, replace the 40-year-old nutcracker!* Annals of Behavioral Medicine, 2008. **35**(2): p. 136-149.

48. Russell, J.A., A. Weiss, and G.A. Mendelsohn, *Affect Grid - a Single-Item Scale of Pleasure and Arousal.* Journal of Personality and Social Psychology, 1989. **57**(3): p. 493-502.

49. Chen, M.J., X. Fan, and S.T. Moe, *Criterion-related validity of the Borg ratings of perceived exertion scale in healthy individuals: a meta-analysis.* J Sports Sci, 2002. **20**(11): p. 873-99.

50. Medicine, A.C.o.S., *ACSM's guidelines for exercise testing and prescription*. 8th ed ed. 2010, Philadelphia: Lippincott, Williams & Wilkins.

51. Cook, D.B., et al., *Naturally occurring muscle pain during exercise: assessment and experimental evidence.* Med Sci Sports Exerc, 1997. **29**(8): p. 999-1012.

52. Thayer, R.E., *Measurement of activation through self-report.* Psychol Rep, 1967. **20**(2): p. 663-78.

53. Ekkekakis, P., et al., *Walking in (affective) circles: can short walks enhance affect?* J Behav Med, 2000. **23**(3): p. 245-75.

54. Sallis, J.F., *The development of self-efficacy scales for health-related diet and exercise behaviors.* Health Education Research, 1988(3): p. 9.

55. Wilson, P.M., et al., *The psychological need satisfaction in exercise scale.* Journal of Sport & Exercise Psychology, 2006. **28**(3): p. 231-251.

56. Mullan, E., D. Markland, and D.K. Ingledew, *A graded conceptualisation of self-determination in the regulation of exercise behaviour: Development of a measure using confirmatory factor analytic procedures.* Personality and Individual Differences, 1997. **23**(5): p. 745-752.

57. Williams, G.C., et al., *Motivational predictors of weight loss and weight-loss maintenance.* J Pers Soc Psychol, 1996. **70**(1): p. 115-26.

58. Subar, A.F., et al., *The Automated Self-Administered 24-hour dietary recall (ASA24): a resource for researchers, clinicians, and educators from the National Cancer Institute.* J Acad Nutr Diet, 2012. **112**(8): p. 1134-7.

59. Conway, J.M., et al., *Effectiveness of the US Department of Agriculture 5-step multiple-pass method in assessing food intake in obese and nonobese women.* Am J Clin Nutr, 2003. **77**(5): p. 1171-8.

APPENDIX 1 (a): Recruitment Flyer

APPENDIX 1 (b): Recruitment Brochure

Screening Application

APPENDIX 2: Screening Application

The Motivation to Exercise

This study is being done to determine motivational factors involved in exercise. The purpose of this study is to research the effects of exercise on motivation levels. We expect to find out whether participants change their motivation to exercise when starting an exercise program.Participants will be enrolled in a six-week exercise program and provided memberships to Choice Health and Fitness. Exercise sessions will be about one hour per day, three days per week and prescribed by a USDA researcher. Three days of baseline assessments will take place assessing body composition, physical fitness, dietary intake, and exercise motivation before the 6-week exercise program begins. Follow-up testing sessions will also take place after the 6-week exercise program. You would visit the Grand Forks Human Nutrition Research Center or Choice Health and Fitness for an information and screening visit where you will be given information about this study and complete further screening to assure that you meet eligibility criteria for participation. If you qualify for and are interested in participating in this study, you will be scheduled for the initial three baseline testing sessions before initiation of the exercise program. This study is being conducted by Dr. James R. Roemmich of the Grand Forks Human Nutrition Research Center in Grand Forks, ND.

If you are interested in joining this study please answer the following questions.

How did you hear about this study?

O UND Newsletters O UND Radio

O Tidbits O City Briefs

O Flyers O Grocery Stores

O Thrift Stores O Retail Stores

O Brochure O Senior Citizens Center

O Library O Choice Health and Fitness Center

O Other:­­­­­­­­­­­­­­­________________________

Gender: Male ____ Female ____

Are you pregnant, lactating, or planning to become pregnant in the next 6 months? ** No____ Yes_____

Are you between 18 and 49 years of age? * No_____ Yes_____

Do you have any chronic medical conditions? No____ Yes_____

If yes, please check the proper box/boxes and explain.

O Diabetes

O Major health problem

Explain:_________________________

O Cardiovascular disease

Explain:_________________________

O Pulmonary disease

Explain:_________________________

O Metabolic disease

Explain:_________________________

O Orthopedic problem that limits exercise

Explain:_________________________

O Other:________________________

Explain:_________________________

Are you taking any medications, vitamins, or supplements? No____ Yes____

If yes, please list.

Do you regularly use products containing tobacco or nicotine? ** No____ Yes_____

Are you on a diet? No____ Yes_____

If yes, please explain

Do you have any medical problems that limit your ability to safely participate in physical activity? ** No____ Yes_____

Do you exercise regularly (planned and structured aerobic activity more than two times per week)? ** No____ Yes_____

Do you have high occupational physical activity (construction trades, farm work, freight mover)?

No____ Yes_____

If yes, please explain

How much do you weigh? ____________lbs.

How tall are you? _______ft. _______in.

Name: ________________________________________

Address: _________________________________________________________

Street Apt #

_________________________________________________________

City/Town State Zip Code

Email Address: _____________________________________________________

Phone Number: _____________________________________________________

Your application is complete. It will be reviewed for study eligibility. We will contact you if an

answer in your application needs to be clarified. You will be told whether or not you qualify for

the study.

If you qualify for the study, you will be invited to meet with Dr. Roemmich or his designee. They will discuss with you the study requirements and answer any questions you may have. During this meeting you will complete a questionnaire which will provide information about your general health and your medication and supplement use. Your height and weight will also be measured at this time.

If you have questions, please contact Bill Siders at 701 795-8430 or Dr. Kyle Flack at 701 795-8430 or e-mail your questions to [william.siders@ars.usda.gov](mailto:william.siders@ars.usda.gov) or [kyle.flack@ars.usda.gov](mailto:kyle.flack@ars.usda.gov). Thank you.

* If YES, the screen goes to the next question. If NO, the screen shows the message “Thank

you for your interest in joining the “Compensatory Response” study. Unfortunately, you are not

eligible for this study.

** If NO, the screen goes to the next question. If YES, the screen shows the message “Thank

you for your interest in joining the “Compensatory Response” study. Unfortunately, you are not eligible for this study.

**INFORMED CONSENT**

APPENDIX 3: Informed consent

**TITLE:** The Motivation to Exercise

**PROJECT DIRECTOR:** Dr. James N. Roemmich

**PHONE #** 701-795-8272

**DEPARTMENT:** Grand Forks Human Nutrition Research Center

**STATEMENT OF RESEARCH**

A person who is to participate in the research must give his or her informed consent to such participation. This consent must be based on an understanding of the nature and risks of the research. This document provides information that is important for this understanding. Research projects include only subjects who choose to take part. Please take your time in making your decision as to whether to participate. If you have questions at any time, please ask.

**WHAT IS THE PURPOSE OF THIS STUDY?**

You are invited to be in a research study investigating the motivational factors involved in exercise. The purpose of this study is to research the effects of exercise on motivating factors implicated in physical activity and behavior. We expect to find out whether subjects change their levels of motivation and activity behaviors when starting an exercise program.

**HOW MANY PEOPLE WILL PARTICIPATE?**

Approximately 90 individuals will take part in this study at the Grand Forks Human Nutrition Research Center (GFHNRC).

**ELIGIBILITY:** You may participate if you are between the ages of 18 and 49 years with a body mass index (BMI) of 19-35 kg/m^2^. You must not be currently dieting to lose weight or exercising more than once per week on a regular basis.

You cannot participate in the study if you:

- are taking any medications that affect energy expenditure or eating
- have not gained or lost more than 10 pounds over the past 3 months
- use tobacco
- are pregnant or lactating or plan to become pregnant in the next 6 months
- have any medical conditions that prevent you from safely participating in physical activity
- have high occupational activity such as construction trades, farm workers, and freight movers who complete most of their work by hand

**HOW LONG WILL I BE IN THIS STUDY?**

Your participation in the study involves attending an informational and screening visit (about 2 hours). If you qualify for the study and agree to participate, you will visit Choice Health and Fitness Center (CHFC) or the Grand Forks Human Nutrition Research Center (GFHNRC) for 3 baseline visits on separate days, each lasting from 1-2 hours. Then you will start a prescribed exercise program for about 1 hour each day, 3 days a week, for 6 weeks. After 6 weeks you will again visit CHFC or GFHNRC on 3 separate days for 1-2 hours each. One month after the study you will return for additional follow-up visits.

**WHAT WILL HAPPEN DURING THIS STUDY?**

***Screening visit:*** Dr. Roemmich or his designee will tell you about the study and answer any questions you may have. If you want to join the study, you will be asked to provide written informed consent. After signing this consent, you will be asked to fill out a demographic form which will be used to describe the group characteristics, a W-9 which is required before a check can be issued for payment, a medical history form to determine if you are medically able to participate, and other questionnaires to determine whether you meet the study entry criteria. You will be provided an activity monitor to wear during all awake hours for one week to record physical activity. Your height and weight will be measured and you will be scheduled for your 3 baseline visits.

***Baseline Measurements:***

A variety of measurements will be completed during the 3 baseline visits:

- Visit 1 (you must arrive 2-3 hours after eating):
  - You will complete questionnaires and computer activities to determine how much you like and value physical activity (60 min).
  - Using a computer program at home, you will report everything you ate for 2 weekdays and 1 weekend day (30 minutes each day).
- Visit 2 (you must arrive at least 2 hours after eating):
  - Your body fat will be measured using a very low-dose x-ray machine (15 min)
  - Weight using a scale (1 min).
  - You will complete a moderate to hard intensity fitness test while walking on a treadmill. You will wear a tight-fitting face mask during the test, in addition to an activity monitor and/or chest strap to measure energy expended (60 min including rest and exercise time).
- Visit 3 (you must arrive at least 2 hours after eating):
  - You will be guided through a 15-minute exercise session on a treadmill and complete questionnaires before, during, and after exercising (45 min).

***Treatment Procedure:***

You will be randomly assigned to one of two exercise groups during this study or to the control group. You will not have a choice as to which group you are in. A membership to CHFC will be provided to you at no cost. One exercise group will expend 300 calories per day and the other group will expend 150 calories per day while exercising. This exercise will take about 30 to 60 minutes to complete. Most of the exercise sessions will be on your own schedule, but the first 3-5 sessions will be supervised by trained research staff to introduce you to the exercise equipment and instruct you on the intensity and length of exercise needed to reach your goal. Then, sessions will be supervised as needed to encourage you to adhere to the program. If you miss a session, you will have to make it up on another day. You will be asked to follow our exercise program for 6 weeks. For the four weeks after that you can exercise however you want. The control group will receive the exercise program and membership to CHFC after completing the 10 week study. During the first 6 weeks, you will complete the following measurements:

- Energy expended during exercise - during all exercise sessions you will wear an activity monitor and/or chest strap to measure the energy expended during exercise. The monitors transmit to a small display that you will use to make sure that you exercising at your prescribed intensity and amount.
- After 6 weeks: you will follow the baseline assessment procedure and schedule.
- One month after the conclusion of the intervention (week 10): you will follow the baseline assessment procedure and schedule.

You will continue participating in the exercise sessions until all of the testing has been completed.

**WHAT ARE THE RISKS OF THE STUDY?**

There may be some risk from being in this study.

***Questionnaires:*** You may experience frustration when completing surveys. Some questions may be of a sensitive nature, and you may therefore become upset as a result. If, however, you become upset by questions, you may stop at any time or choose not to answer a question.

***Exercise and Fitness Tests:*** There is a small risk of sprains, strains, and broken bones as the result of any exercise. To reduce this risk, you will be supervised for the first sessions by experienced personnel. Some soreness may occur 24-48 hours after exercise but this will go away with time. Exercise can uncover or worsen hidden heart problems such as not enough blood flow to the heart muscle and irregular beats. It is unlikely you will have problems with your heart or circulatory system. Should you develop symptoms of any medical problems, testing will be stopped immediately.

***DXA Scan***: The DXA scan is an x-ray and is considered to be a no greater than minimal risk procedure. The radiation dose of the whole-body scan is no more than 1.0 millirem. This dose is equivalent to approximately 1/620 of normal annual background radiation, ¼ of the radiation received in a long flight, or 1/10 of the radiation received in a chest x-ray. A quality assurance check will be completed on the DXA each day prior to its use; the software will not allow the use of the DXA if the quality assurance check fails. Each subject will receive two DXA scans, and an extra scan may be needed. The effects of small doses of radiation on a developing fetus are not known; therefore, we will not knowingly allow a pregnant woman to have a DXA. Pregnancy tests will be done before the DXA if you are a woman of child-bearing age.

***Armbands and chest straps:*** There is a possibility of skin irritation from the armband and the chest strap. These devices will be cleaned thoroughly between uses, and trained staff will instruct you on how to properly wear the devices. If any discomfort occurs, please report it to the research staff immediately and you will be instructed to remove the device or adjust its position.

**WHAT ARE THE BENEFITS OF THIS STUDY?**

You may not benefit personally from being in this study. However, we hope that, in the future, other people might benefit from this study because we hope to gain insight into what causes people to change their physical activity and eating habits when they start an exercise program. This knowledge could improve weight-loss efforts in the future.

**WILL IT COST ME ANYTHING TO BE IN THIS STUDY?**

You will not have any costs for being in this research study.

**WILL I BE PAID FOR PARTICIPATING?**

You will be paid for being in this research study. In addition to a 6 month membership to Choice Health & Fitness Center, you will receive reimbursement in the amount of $500 for completion of the study. There is no reimbursement for screening procedures. You will receive $150 for completing the baseline measures, $50 for completing the first two weeks of exercise, and $300 for completing the end of the exercise training and testing visits. You will be required to provide your SSN and address to receive your payment check (W9 form).

**WHO IS FUNDING THE STUDY?**

There will be no independent funding for this research study. The USDA ARS-GFHNRC will provide funds to support the activities that are required to conduct the study. No one on the research team will receive a direct payment or an increase in salary for conducting this study.

**CONFIDENTIALITY**

The records of this study will be kept private to the extent permitted by law. In any report about this study that might be published, you will not be identified. None of the study results will have any names attached. Confidentiality will be maintained by assigning an identification number which will be used to anonymously code your research data for computer entry. This consent and the check information will be kept in a locked file at the Grand Forks Human Nutrition Research Center. Dr. James Roemmich and the staff assigned to the research study will have access to the data. Confidential information may be made available to the US Department of Agriculture as specified in the USDA/ARS Privacy Act System of Records and to the University of North Dakota and as required by law or court order. Clinical trial information will be submitted to the National Institutes of Health/National Library of Medicine to be included in the clinical trials registry data bank (www.clinicaltrials.gov)

We will monitor you by video camera while you complete the computer activities used to determine how much you are motivated for food and physical activity, but you will not be recorded at any time during the study.

**COMPENSATION FOR INJURY**

In the event that this research activity results in an injury, treatment will be available including first aid, emergency treatment and follow-up care as needed. Payment for any such treatment is to be provided by you (you will be billed) or your third-party payer, if any (such as health insurance, Medicare, etc.) No funds have been set aside to compensate you in the event of injury. Also, the study staff cannot be responsible if you knowingly and willingly disregard the directions they give you.

**IS THIS STUDY VOLUNTARY?**

Your participation is voluntary. You may choose not to participate or you may discontinue your participation at any time without penalty or loss of benefits to which you are otherwise entitled. Your decision whether or not to participate will not affect your current or future relations with the University of North Dakota or the Grand Forks Human Nutrition Research Center.

If you decide to leave the study early, we ask that you call Bill Siders at 701-795-8430 or Kyle Flack at 701-795-8342 to inform them of your withdrawal.

You will be informed by the research investigator of this study of any significant new findings that develop during the study which may influence your willingness to continue to participate in the study.

There may be special circumstances that will result in your early withdrawal from the study without your approval. Such circumstances include realization that exercise is not healthy for you or realization that other conditions might make continued participation harmful to you. You will also be withdrawn from the study if you are not willing or not able to follow the study directions.

**CONTACTS AND QUESTIONS?**

The researchers conducting this study are Dr. James Roemmich and Dr. Kyle Flack. You may ask any questions you have now. If you later have questions, concerns, or complaints about the research please contact Bill Siders at 701-795-8430 or Dr. Flack at 701-795-8342 during the day or the Metabolic Unit at 701-795-8339 after hours.

If you have questions regarding your rights as a research subject, or if you have any concerns or complaints about the research, you may contact the University of North Dakota Institutional Review Board at (701) 777-4279. Please call this number if you cannot reach research staff, or you wish to talk with someone else.

Your signature indicates that this research study has been explained to you, that your questions have been answered, and that you agree to take part in this study. You will receive a copy of this form.

Subjects Name: ______________________________________________________

__________________________________ ______________­­_____

Signature of Subject Date

I have discussed the above points with the subject or, where appropriate, with the subject’s legally authorized representative.

__________________________________ ___________________

Signature of Person Who Obtained Consent Date


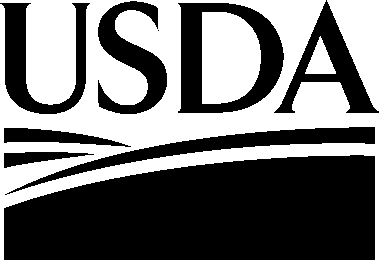
 ***Grand Forks Human Nutrition Research Center USDA-ARS***

***2420 2nd Avenue North STOP 9034***

APPENDIX 4: Demographic Information

***Grand Forks, North Dakota 58202 - 9034***

***Telephone: (701) 795-8353***

**Demographic Information**

**#1. Name:**

***(last) (first) (middle initial)***

**#2. Date Of Birth: (*mm/dd/yyyy*)**

**#3. Gender: Male Female**

**#4. Race (Select Only One): American Indian or Alaskan Native**

**Asian**

**Black or African American**

**Native Hawaiian or Other Pacific Islander**

**White**

**Multi-racial**

**Unknown**

**I prefer not to answer**

**#5. Ethnicity (Select Only One): Hispanic or Latino**

**Not Hispanic or Latino**

**Unknown**

**I prefer not to answer**

**#6. Mailing Address:**

**#7. *Preferred* method of Contact: _ E-mail Cell Phone Text**

**#8. E-mail Address:**

**#9. Cell Phone Number:**

**#10. Cell Phone Carrier:**

APPENDIX 5: Health History

# Health History Questionnaire

NAME: ___________________________ DATE: __________________________

Birthdate: ______________________________Gender: Male Female

Height:_____________________ Weight:________________ BMI_______

Person to contact in case of Emergency:

Name: _______________________________ Phone: ______________________

1. Are you taking any medications or drugs?

If YES, please list medication, dose and reason: Medication:_________________Dose:_____________Reason:_______________ Medication:_________________Dose:_____________Reason:_______________

Medication:_________________Dose:_____________Reason:_______________

Medication:_________________Dose:_____________Reason:_______________

*Use the back for more space*

YES NO

□ □ 1. Has your doctor ever said that you have a heart condition and that you should only do

physical activity recommended by a doctor?

□ □ 2. Do you feel pain in your chest when you do physical activity?

□ □ 3. In the past month, have you had chest pain when you were not doing physical activity?

□ □ 4. Do you lose your balance because of dizziness or do you ever lose consciousness?

□ □ 5. Do you have a bone or joint problem that could be made worse by a change in your

physical activity?

□ □ 6. Is your doctor currently prescribing drugs (for example, water pills) for your blood

pressure or heart condition?

□ □ 7. Do you know of any other reason why you should not do physical activity?

| **General Health Questions:** | **Yes** |  | **No** |
| --- | --- | --- | --- |
| **History of heart problems, chest pain or stroke** |  |  |  |
| **Increased blood pressure** |  |  |  |
| **History of breathing or lung problems** |  |  |  |
| **Diabetes or thyroid disease** |  |  |  |
| **Seizures, tremors or severe headaches** |  |  |  |
| **Arthritis or other bone problems** |  |  |  |
| **Increased blood cholesterol** |  |  |  |
| **Use products containing tobacco or nicotine regularly** |  |  |  |
| **Recent surgery (last 12 months)** |  |  |  |
| **Any other chronic illness or condition Please List:** |  |  |  |

## Comments:

participant id ____________________ date _______________

APPENDIX 6: PRETIE-Q

**Preference for and Tolerance of**

**the Intensity of Exercise Questionnaire (PRETIE-Q)**

*Please, read each of the following statements and then use the response scale below to indicate whether you agree or disagree with each statement. There are no right or wrong answers. Work quickly and circle the number that best represents what you believe and how you feel.*

*I totally disagree I disagree I neither agree or disagree I agree I totally agree*

*( 1 ) ( 2 ) ( 3 ) ( 4 ) ( 5 )*

1 Feeling tired during exercise is my signal to slow down or stop. *1 2 3 4 5*

2 I would rather work out at low intensity levels for a long duration

than at high intensity levels for a short duration. *1 2 3 4 5*

3 During exercise, if my muscles begin to burn excessively or if I find

myself breathing very hard, it is time for me to ease off. *1 2 3 4 5*

4 I’d rather go slow during my workout, even if that means taking more time. *1 2 3 4 5*

5 While exercising, I try to keep going even after I feel exhausted. *1 2 3 4 5*

6 I would rather have a short, intense workout than a long, low-intensity

workout. *1 2 3 4 5*

7 I block out the feeling of fatigue when exercising. *1 2 3 4 5*

8 When I exercise, I usually prefer a slow, steady pace. *1 2 3 4 5*

9 I’d rather slowdown or stop when a workout starts to get too tough. *1 2 3 4 5*

10 Exercising at a low intensity does not appeal to me at all. *1 2 3 4 5*

11 Fatigue is the last thing that affects when I stop a workout;

I have a goal and stop only when I reach it. *1 2 3 4 5*

12 While exercising, I prefer activities that are slow-paced and do not

require much exertion. *1 2 3 4 5*

13 When my muscles start burning during exercise, I usually ease off some. *1 2 3 4 5*

14 The faster and harder the workout, the more pleasant I feel. *1 2 3 4 5*

15 I always push through muscle soreness and fatigue when workingout. *1 2 3 4 5*

16 Low-intensity exercise is boring. *1 2 3 4 5*

APPENDIX 7: Discomfort Intolerance Scale

Participant id ____________________ date _______________

**Discomfort Intolerance Scale (DIS)***

*Please rate the statements (1 – 7) on a scale ranging from 0 (not at all like me) to 6 (extremely*

*like me) by circling the number that best reflects how the rating describes you.*

*not at all extremely*

*like me like me*

1 I can tolerate a great deal of

physical discomfort *0 1 2 3 4 5 6*

2 I have a high pain threshold *0 1 2 3 4 5 6*

3 I take extreme measures to avoid

feeling physically uncomfortable *0 1 2 3 4 5 6*

4 I’m the kind of person who never

takes medication, like aspirin,

when I have aches and pains *0 1 2 3 4 5 6*

5 I push my physical limits when I

exercise *0 1 2 3 4 5 6*

6 When I begin to feel physically

uncomfortable, I quickly take

steps to relieve the discomfort *0 1 2 3 4 5 6*

7 I am more sensitive to feeling

discomfort compared to most

persons *0 1 2 3 4 5 6*

APPENDIX 8: Social Support and Exercise

Participant id ____________________ date _______________

SOCIAL SUPPORT AND EXERCISE SURVEY

Below is a list of things people might do or say to someone who is trying to exercise regularly. If you are not trying to exercise, then some of the questions may not apply to you, but please read and give an answer to every question.

Please rate each question twice. Under family, rate how often anyone living in your household has said or done what is described during the last three months. Under friends, rate how often your friends, acquaintances, or coworkers have said or done what is described during the last three months.

Please write one number from the following rating scale in each space:

none

rarely

a few times

often

very often

does not apply

1 2 3 4 5 8

During the past three months, my family (or members of my household) or friends:

Family Friends

11. Exercised with me. 11. __11.

12.

13.

Offered to exercise with me.

Gave me helpful reminders to exercise ("Are you going to exercise tonight?”).

12.

13.

12.

_13.

14.

Gave me encouragement. to stick with my exercise program. 14. _ 14.

15.

Changed their schedule so we could exercise together.

15.

15.

16.

Discussed exercise with me.

16. 16.

17.

Complained about the time I spend exercising. 17.

17.

18.

Criticized me or made fun of me for exercising. 18. 18.

19.

20.

Gave me rewards for exercising (bought me something or gave me something I like).

Planned for exercise on recreational outings.

19. 19.

20. 20.

21.

Helped plan activities around my exercise.

21.

21._

22.

Asked me for ideas on how they can get more exercise. 22. _22.

23. Talked about how much they like to exercise.

23.

23._

Participant id ____________________ date _______________

APPENDIX 9: Exercise Self-Efficacy Scale

Exercise Self-Efficacy Scale

The items listed below are designed to assess your beliefs in your ability to continue exercising on a three time per week basis at moderate intensities (upper end of your perceived exertion range), for **40+** minutes per session in the future. Using the scales listed below please indicate how confident you are that you will be able to continue to exercise in the future.

For example, if you have complete confidence that you could exercise three times per week at moderate intensity for **40+** minutes for the next four weeks without quitting, you would **circle 100%**. However, if you had no confidence at all that you could exercise at your exercise prescription for the next four weeks without quitting, (that is, confident you would not exercise), you would **circle 0%**.

Please remember to answer honestly and accurately. There are no right or wrong answers.

**Mark your answer by circling a %:**

0% 10% 20% 30% 40% 50% 60% 70% 80% 90% 100%

NOT AT ALL MODERATELY HIGHLY

CONFIDENT CONFIDENT CONFIDENT

1. I am able to continue to exercise three times per week at moderate intensity, for

40+ minutes without quitting for the NEXT WEEK

0% 10% 20% 30% 40% 50% 60% 70% 80% 90% 100%

2. I am able to continue to exercise three times per week at moderate intensity, for

40+ minutes without quitting for the NEXT TWO WEEKS

0% 10% 20% 30% 40% 50% 60% 70% 80% 90% 100%

3. I am able to continue to exercise three times per week at moderate intensity, for

40+ minutes without quitting for the NEXT THREE WEEKS

0% 10% 20% 30% 40% 50% 60% 70% 80% 90% 100%

4. I am able to continue to exercise three times per week at moderate intensity, for

40+ minutes without quitting for the NEXT FOUR WEEKS

0% 10% 20% 30% 40% 50% 60% 70% 80% 90% 100%

5. I am able to continue to exercise three times per week at moderate intensity, for

40+ minutes without quitting for the NEXT FIVE WEEKS

0% 10% 20% 30% 40% 50% 60% 70% 80% 90% 100%

6. I am able to continue to exercise three times per week at moderate intensity, for

40+ minutes without quitting for the NEXT SIX WEEKS

0% 10% 20% 30% 40% 50% 60% 70% 80% 90% 100%

7. I am able to continue to exercise three times per week at moderate intensity, for

40+ minutes without quitting for the NEXT SEVEN WEEKS

0% 10% 20% 30% 40% 50% 60% 70% 80% 90% 100%

8. I am able to continue to exercise three times per week at moderate intensity, for

40+ minutes without quitting for the NEXT EIGHT WEEKS

0% 10% 20% 30% 40% 50% 60% 70% 80% 90% 100%

Participant id ____________________ date _______________

APPENDIX 10: Exercise Confidence Survey

Exercise Confidence Survey

**EXERCISE CONFIDENCE SURVEY**

Below is a list of things people might do while trying to increase or continue regular exercise. We are interested in exercises like running, swimming, brisk walking, bicycle riding, or aerobics classes.

Whether you exercise or not, please rate how confident you are that you could really motivate yourself to do things like these consistently, for at least six months.

Please circle one number for each question.

How sure are you that you can do these things?

I know I Maybe I I know I Does not

cannot can can apply

1. Get up early, even on weekends, to exercise. 1 2 3 4 5 -

2. Stick to your exercise program after a long, tiring day 1 2 3 4 5 -

at work.

3. Exercise even though you are feeling depressed. 1 2 3 4 5 -

4. Set aside time for a physical activity program; that is, 1 2 3 4 5 -

walking, jogging. swimming, biking, or other continuous

activities for at least 30 minutes, 3 times per week.

5. Continue to exercise with others even though they 1 2 3 4 5 -

seem too fast or too slow for you.

6. Stick to your exercise program when undergoing a 1 2 3 4 5 -

stressful life change (e.g., divorce, death in the family,

moving).

7. Attend a party only after exercising. 1 2 3 4 5 -

8. Stick to your exercise program when your family is 1 2 3 4 5 -

demanding more time from you.

9. Stick to your exercise program when you have) 1 2 3 4 5 -

household chores to attend to.

10. Stick to your exercise program even when you have 1 2 3 4 5 -

excessive demands at work.

11. Stick to your exercise program when social obligations 1 2 3 4 5 -

are very time consuming.

12. Read or study less in order to exercise more. 1 2 3 4 5 -

APPENDIX 11: PNSE

Psychological Need Satisfaction in Exercise Scale

Participant id ____________________ date _______________

The following statements represent different experiences people have when they exercise. Please answer the following questions by considering how YOU TYPICALLY feel while you are exercising.

More False than true

More True

than false

Mostly True

True

False

Mostly False

| 1. | I feel that I am able to complete exercises that are personally challenging | 1 | 2 | 3 | 4 | 5 | 6 |
| --- | --- | --- | --- | --- | --- | --- | --- |
| 2. | I feel attached to my exercise companions because they accept me for who I am | 1 | 2 | 3 | 4 | 5 | 6 |
| 3. | I feel like I share a common bond with people who are important to me when we exercise together | 1 | 2 | 3 | 4 | 5 | 6 |
| 4. | I feel confident I can do even the most challenging exercises | 1 | 2 | 3 | 4 | 5 | 6 |
| 5. | I feel a sense of camaraderie with my exercise companions because we exercise for the same reasons | 1 | 2 | 3 | 4 | 5 | 6 |
| 6. | I feel confident in my ability to perform exercises that personally challenge me | 1 | 2 | 3 | 4 | 5 | 6 |
| 7. | I feel close to my exercise companions who appreciate how difficult exercise can be | 1 | 2 | 3 | 4 | 5 | 6 |
| 8. | I feel free to exercise in my own way | 1 | 2 | 3 | 4 | 5 | 6 |
| 9. | I feel free to make my own exercis e program decisions | 1 | 2 | 3 | 4 | 5 | 6 |
| 10. | I feel capable of completing exercises that are challenging to me | 1 | 2 | 3 | 4 | 5 | 6 |
| 11. | I feel like I am in charge of my exercise program decisions | 1 | 2 | 3 | 4 | 5 | 6 |
| 12. | I feel like I am capable of doing even the most challenging exercises | 1 | 2 | 3 | 4 | 5 | 6 |
| 13. | I feel like I have a say in choosing the exercises that I do | 1 | 2 | 3 | 4 | 5 | 6 |
| 14. | I feel connected to the people who I interact with while we exercise together | 1 | 2 | 3 | 4 | 5 | 6 |
| 15. | I feel good about the way I am able to complete challenging exercises | 1 | 2 | 3 | 4 | 5 | 6 |
| 16. | I feel like I get along well with other people who I interact with while we exercise together | 1 | 2 | 3 | 4 | 5 | 6 |
| 17. | I feel free to choose which exercises I participate in | 1 | 2 | 3 | 4 | 5 | 6 |
| 18. | I feel like I am the one who decides what exercises I do | 1 | 2 | 3 | 4 | 5 | 6 |

EXERCISE REGULATIONS QUESTIONNAIRE (BREQ-3)

APPENDIX 12: BREQ-3

Subject ID: _______ Date___________

*Why do you engage in exercise?*

We are interested in the reasons underlying peoples’ decisions to engage or not engage in physical exercise. Using the scale below, please indicate to what extent each of the following items is true for you. Please note that there are no right or wrong answers and no trick questions. We simply want to know how you personally feel about exercise. Your responses will be held in confidence and only used for our research purposes.

Not true sometimes Very true
 for me true for me for me

1 It’s important to me to exercise regularly 0 1 2 3 4

2 I don’t see why I should have to exercise 0 1 2 3 4

3 I exercise because it’s fun 0 1 2 3 4

4 I feel guilty when I don’t exercise 0 1 2 3 4

5 I exercise because it is consistent with my life goals 0 1 2 3 4

6 I exercise because other people say I should 0 1 2 3 4

7 I value the benefits of exercise 0 1 2 3 4

8 I can’t see why I should bother exercising 0 1 2 3 4

9 I enjoy my exercise sessions 0 1 2 3 4

10 I feel ashamed when I miss an exercise session 0 1 2 3 4

11 I consider exercise part of my identity 0 1 2 3 4

12 I take part in exercise because my 0 1 2 3 4
 friends/family/partner say I should

13 I think it is important to make the effort to 0 1 2 3 4

exercise regularly

14 I don’t see the point in exercising 0 1 2 3 4

15 I find exercise a pleasurable activity 0 1 2 3 4

16 I feel like a failure when I haven’t exercised 0 1 2 3 4

in a while

17 I consider exercise a fundamental part of who I am 0 1 2 3 4

18 I exercise because others will not be 0 1 2 3 4
 pleased with me if I don’t

19 I get restless if I don’t exercise regularly 0 1 2 3 4

20 I think exercising is a waste of time 0 1 2 3 4

21 I get pleasure and satisfaction from 0 1 2 3 4

participating in exercise

22 I would feel bad about myself if I 0 1 2 3 4

was not making time to exercise

23 I consider exercise consistent with my values 0 1 2 3 4

24 I feel under pressure from my 0 1 2 3 4

friends/family to exercise

**LIKING OF PHYSICAL ACTIVITY CHOICES**

APPENDIX 13: Liking of PA and sedentary choices

Date________________ Participant #______________________

Please circle the number that best represents how much you like each activity listed.

**Elliptical**

**1 2 3 4 5 6 7 8 9 10**

*Do not like it at all Like it very much*

**Treadmill**

**1 2 3 4 5 6 7 8 9 10**

*Do not like it at all Like it very much*

**Stationary Bike**

**1 2 3 4 5 6 7 8 9 10**

*Do not like it at all Like it very much*

**LIKING OF SEDENTARY CHOICES**

Date________________ Participant #______________________

Please circle the number that best represents how much you like each activity listed.

**Reading magazines**

**1 2 3 4 5 6 7 8 9 10**

*Do not like it at all Like it very much*

**Word Games**

**1 2 3 4 5 6 7 8 9 10**

*Do not like it at all Like it very much*

**Puzzles**

**1 2 3 4 5 6 7 8 9 10**

*Do not like it at all Like it very much*

**Pre-Treatment Assessment Form**

APPENDIX 14: SETS

**Participant#________ Date:________**

**Instructions:** The following questions are about a treatment you will soon receive. We want to know how you think you will respond to that treatment. Please indicate how much you agree with each statement by filling in the appropriate circle. For example, if you *strongly disagree* with a statement, fill in the circle on the far left. If you *strongly agree* with a statement, fill in the circle on the far right.

Your answers will be kept confidential, and will not be seen by the clinicians involved in your treatment. Your responses to these questions will not affect the treatment you receive in any way. We realize it may be difficult for you to guess how you will respond to a new treatment. If you are unsure about any statement, please give the bestguess you can. There are no right or wrong answers.

|  | Strongly Disagree | Moderately Disagree | Slightly Disagree | Neither Agree nor Disagree | Slightly Agree | Moderately Agree | Strongly Agree |
| --- | --- | --- | --- | --- | --- | --- | --- |
| This Treatment will be completely effective | ⃝ | ⃝ | ⃝ | ⃝ | ⃝ | ⃝ | ⃝ |
| I am worried about my treatment | ⃝ | ⃝ | ⃝ | ⃝ | ⃝ | ⃝ | ⃝ |
| My lower physical activity will be completely resolved after the treatment | ⃝ | ⃝ | ⃝ | ⃝ | ⃝ | ⃝ | ⃝ |
| I have fears about increasing my activity level | ⃝ | ⃝ | ⃝ | ⃝ | ⃝ | ⃝ | ⃝ |
| I am nervous about negative effects of this treatment | ⃝ | ⃝ | ⃝ | ⃝ | ⃝ | ⃝ | ⃝ |

What treatment are you going to receive?

What specific benefits (if any) do you expect to receive from this treatment?

What specific harms or negative side-effects {if any) do you think may occur because of this treatment?

Have you ever received this treatment before? ___ Yes ___ No

APPENDIX 15: Feeling Scale

**Feeling Scale (FS)**

(Hardy & Rejeski, 1989)

While participating in exercise, it is common to experience changes in mood. Some individuals find exercise pleasurable, whereas others find it to be unpleasant. Additionally, feeling may fluctuate across time. That is, one might feel good and bad a number of times during exercise. Scientists have developed this scale to measure such responses.

**+5 Very good**

**+4**

**+3 Good**

**+2**

**+1 Fairly good**

**0 Neutral**

**-1 Fairly bad**

**-2**

**-3 Bad**

**-4**

**-5 Very bad**

APPENDIX 16: Felt Arousal Scale

**FELT AROUSAL SCALE (FAS)**

(Svebak & Murgatroyd, 1985)

Estimate here how aroused you actually feel. Do this by circling the appropriate number. By “arousal” we meant how “worked-up” you feel. You might experience high arousal in one of a variety of ways, for example as excitement or anxiety or anger. Low arousal might also be experienced by you in one of a number of different ways, for example as relaxation or boredom or calmness.

**1 LOW AROUSAL**

**2**

**3**

**4**

**5**

**6 HIGH AROUSAL**


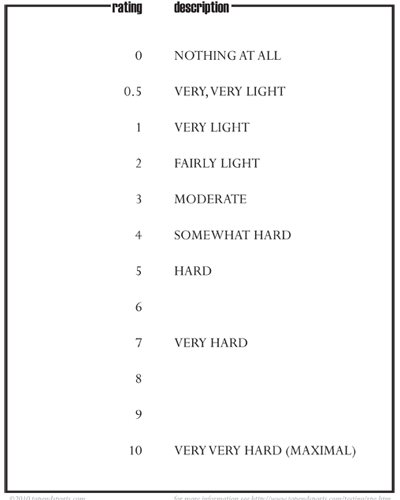


APPENDIX 17: RPE scale

APPENDIX 18: Pain Scale

**Muscle Pain Questionnaire**

Date________________ Participant #______________________

| Category Scale Instructions |
| --- |
|  |
| The numbers on the scale represent a range of pain intensity from “very faint pain” (number 1/2) to “extremely intense pain-almost unbearable” (number 10). When you feel no pain, you should respond with the number 0. If you feel extremely strong pain that is almost unbearable, you should respond with the number 10. If the pain is greater than 10, respond with the number that represents the pain intensity you feel in relation to 10. For example, if the pain is twice as intense as 10 give the number 20. |
| You will be asked to rate the feelings of pain and where it is hurting. When rating these pain sensations be sure to attend only to the specific sensations in your muscles and not report other pains you may be feeling (e.g., seat discomfort). |
| It is very important that your ratings of pain intensity reflect only the degree of hurt you are feeling in your muscles either during exercise or following exercise as pain perceptions are abating. Do not use your ratings as an expression of fatigue (i.e., inability of the muscle to produce force) or relief that the exercise task is completed. |
|  |
|  |
|  |
| In summary, you will be asked to: (i) provide pain intensity ratings in your muscles only; (ii) give ratings as accurately as possible; and (iii) not under- or overestimate the pain, but simply rate your pain honestly.  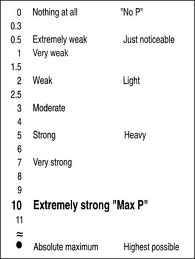 |


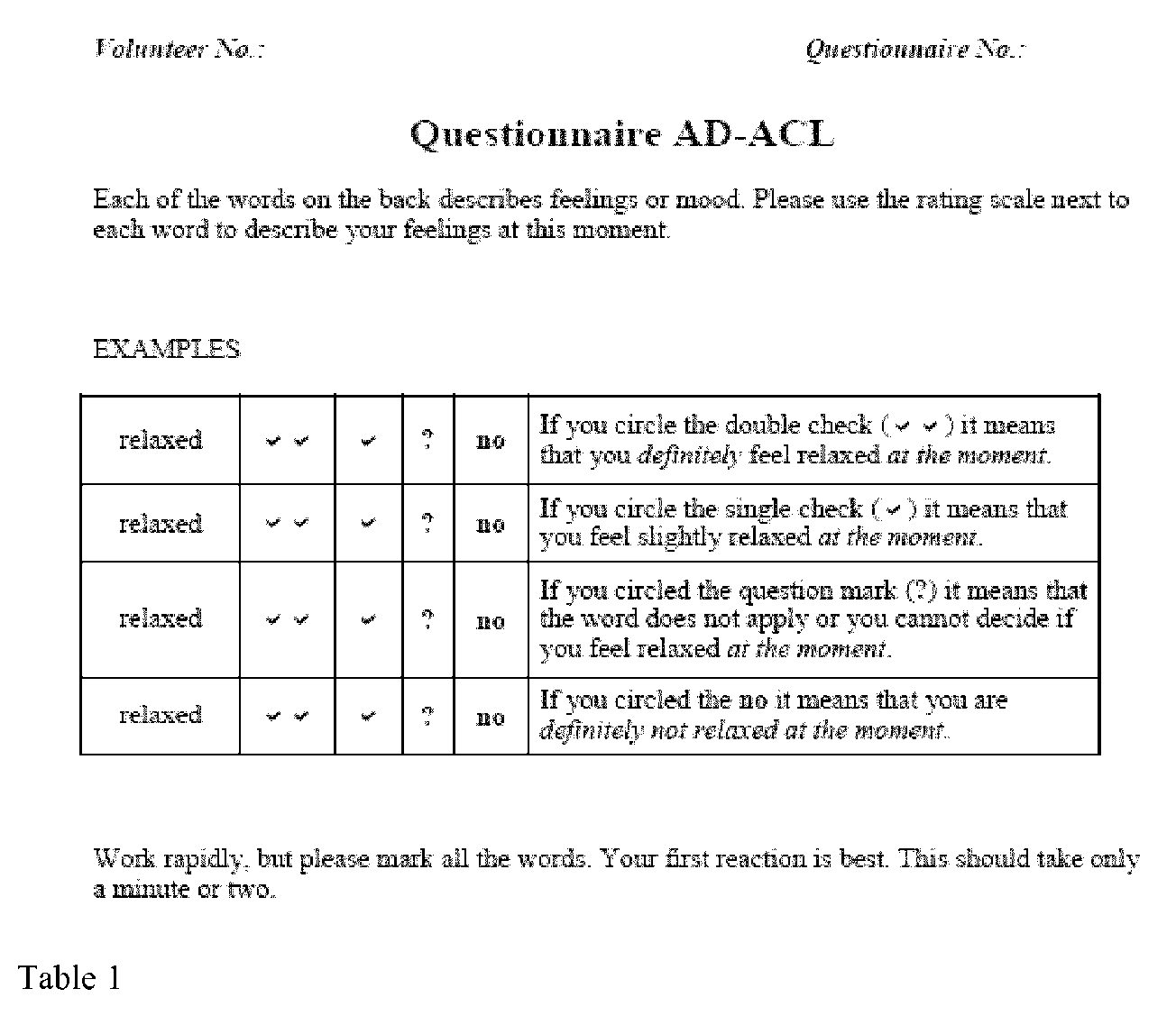


APPENDIX 19: AD ACL


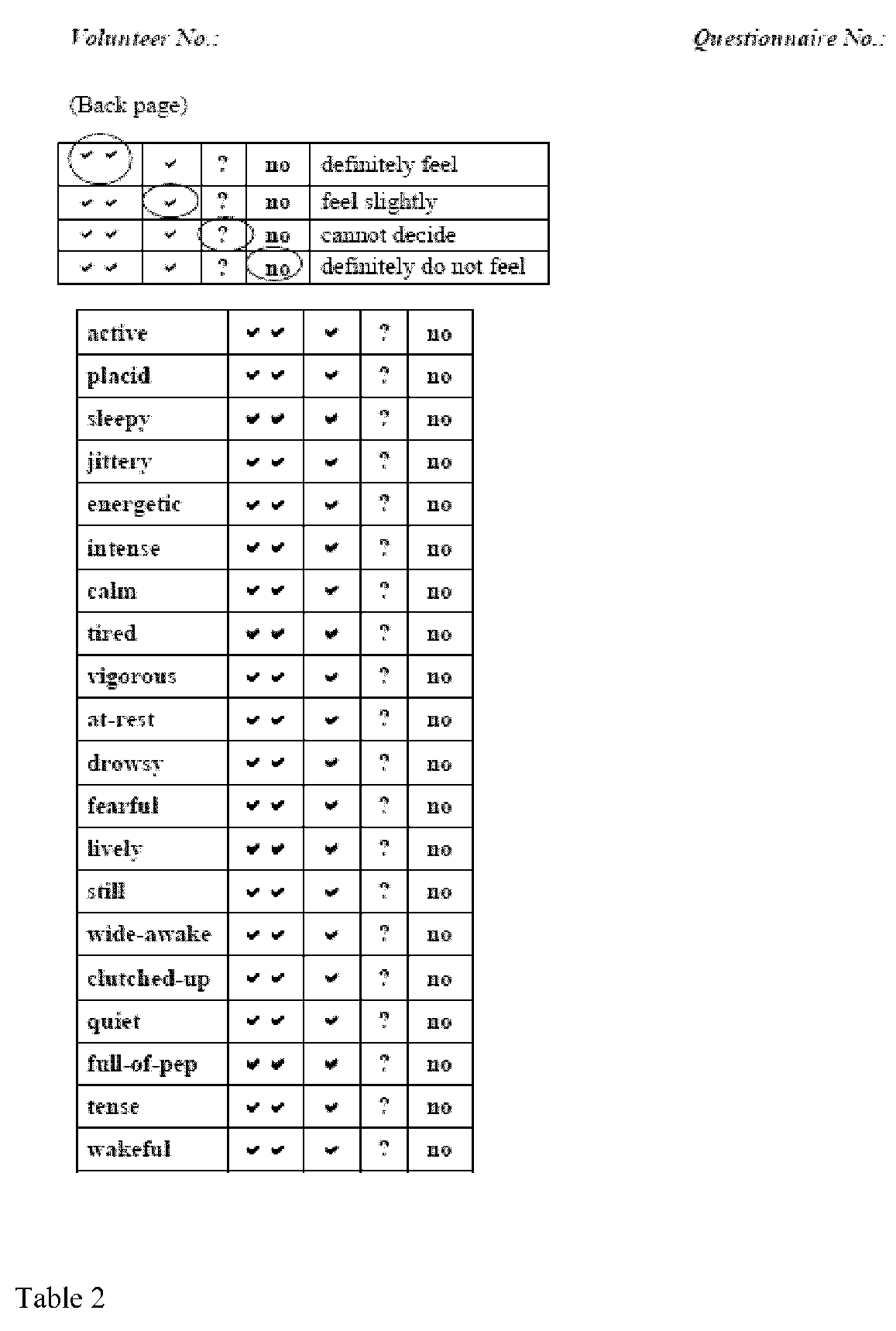


*Today’s Date: ___ /___ /______*

APPENDIX 20: SenseWear monitor log

| SenseWear arm monitor |
| --- |
| Time on: |
| Time off: |
| Time on: |
| Time off: |
| Time on: |
| Time off: |
| Time on: |
| Time off: |

*Today’s Date: ___ /___ /______*

| SenseWear arm monitor |
| --- |
| Time on: |
| Time off: |
| Time on: |
| Time off: |
| Time on: |
| Time off: |
| Time on: |
| Time off: |

*Today’s Date: ___ /___ /______*

| SenseWear arm monitor |
| --- |
| Time on: |
| Time off: |
| Time on: |
| Time off: |
| Time on: |
| Time off: |
| Time on: |
| Time off: |

*Today’s Date: ___ /___ /______*

| SenseWear arm monitor |
| --- |
| Time on: |
| Time off: |
| Time on: |
| Time off: |
| Time on: |
| Time off: |
| Time on: |
| Time off: |

*Today’s Date: ___ /___ /______*

| SenseWear arm monitor |
| --- |
| Time on: |
| Time off: |
| Time on: |
| Time off: |
| Time on: |
| Time off: |
| Time on: |
| Time off: |

*Today’s Date: ___ /___ /______*

| SenseWear arm monitor |
| --- |
| Time on: |
| Time off: |
| Time on: |
| Time off: |
| Time on: |
| Time off: |
| Time on: |
| Time off: |

*Today’s Date: ___ /___ /______*

| SenseWear arm monitor |
| --- |
| Time on: |
| Time off: |
| Time on: |
| Time off: |
| Time on: |
| Time off: |
| Time on: |
| Time off: |

*Today’s Date: ___ /___ /______*

| SenseWear arm monitor |
| --- |
| Time on: |
| Time off: |
| Time on: |
| Time off: |
| Time on: |
| Time off: |
| Time on: |
| Time off: |

**Sport Climate Questionnaire**

APPENDIX 21: Sport Climate

Subject ID ________________ Date______________

This questionnaire contains items that are related to your experience with your coach (trainer). Coaches have different styles in dealing with people, and we would like to know more about how you have felt about your encounters with your coach. Your responses are confidential. Please be honest and candid.

1. I feel that my coach provides me choices and options.

|  |  |  | 1 | 2 | 3 | 4 | 5 | 6 | 7 |
| --- | --- | --- | --- | --- | --- | --- | --- | --- | --- |
|  |  |  | strongly |  |  | neutral |  |  | strongly |
|  |  |  | disagree |  |  |  |  |  | agree |

2. I feel understood by my coach.

1 2 3 4 5 6 7

| strongly | neutral | strongly |
| --- | --- | --- |
| disagree |  | agree |

3. My coach conveyed confidence in my ability to do well at athletics.

1 2 3 4 5 6 7

| strongly | neutral | strongly |
| --- | --- | --- |
| disagree |  | agree |

4. My coach encouraged me to ask questions.

1 2 3 4 5 6 7

| strongly | neutral | strongly |
| --- | --- | --- |
| disagree |  | agree |

5. My coach listens to how I would like to do things.

1 2 3 4 5 6 7

| strongly | neutral | strongly |
| --- | --- | --- |
| disagree |  | agree |

6. My coach tries to understand how I see things before suggesting a new way to do things.

1 2 3 4 5 6 7

| strongly | neutral | strongly |
| --- | --- | --- |
| disagree |  | agree |
