# Supplementary material for: Inducing incentive sensitization of exercise reinforcement among adults who do not regularly exercise—A randomized controlled trial
Source: PLoS One. 2019 May 7;14(5):e0216355. doi: 10.1371/journal.pone.0216355 (PMC6504040; doi:10.1371/journal.pone.0216355)
Supplement: S2 File — Informed consent. (PDF) [file pone.0216355.s003.pdf]

INFORMED CONSENT

**TITLE:**	The Motivation to Exercise
**PROJECT DIRECTOR:**	Dr. James N. Roemmich
**PHONE #**	701-795-8272
**DEPARTMENT:**	Grand Forks Human Nutrition Research Center

STATEMENT OF RESEARCH

A person who is to join in research must give his or her informed consent to such involvement. This consent must be based on knowing the nature and risks of the research. Here we provide information that is important for this understanding. Research projects include only subjects who choose to take part. Please take your time in making your decision as to whether to participate. If you have questions at any time, please ask.

WHAT IS THE PURPOSE OF THIS STUDY?

You are invited to be in a research study of how to make exercise more motivating. The purpose of this study is to understand how we can make exercise more attractive to people. We expect to find out whether subjects change their how motivating they find exercise.

HOW MANY PEOPLE WILL PARTICIPATE?

About 90 participants will take part in this study at the Grand Forks Human Nutrition Research Center (GFHNRC).

ELIGIBILITY: You may join if you are 18 and 49 years old and have a body mass index (BMI) of 19-35 kg/m^2. You must not be dieting to lose weight or exercising more than twice per week. To know if you are eligible, you will complete some questionnaires and other forms. This information may be reviewed by the study's medical doctor to help decide your eligibility.

You <u>cannot</u> join in the study if you:

- are taking any medications that affect energy expenditure or eating
- have gained or lost more than 10 pounds over the past 3 months
- use tobacco
- are pregnant or lactating or plan to become pregnant in the next 6 months

Approval Date: JAN 6 2017
Expiration Date: JAN 5 2018
University of North Dakota IRB

Date _______
Subject Initials _______

- have any medical conditions that prevent you from safely joining in physical activity
- have high work-related activity such as construction and farm work

HOW LONG WILL I BE IN THIS STUDY?

Your involvement in the study involves attending a screening visit (about 2 hours). If you qualify for the study and agree to join, you will visit Choice Health and Fitness Center or the Grand Forks Human Nutrition Research Center for 3 baseline visits on separate days, each lasting from 1-2 hours. Then you will start an exercise program for about 1 hour each day, 3 days a week, for 6 weeks. After 6 weeks you will again visit Choice Health and Fitness or the Grand Forks Human Nutrition Research Center on 3 separate days for 1-2 hours each. One month after the study you will return for the same visits.

WHAT WILL HAPPEN DURING THIS STUDY?

Screening visit: Dr. Roemmich or another researcher will tell you about the study and answer any questions you may have. If you want to join the study, you will be asked to provide written informed consent. After signing this consent, you will be asked to fill out a form which will be used to describe the group characteristics and a W-9 which is required before a check can be issued for payment. You will also fill-out a medical history form, exercise questions, and other forms to determine whether you meet the study entry criteria. You will be provided an activity monitor to wear during all hours awake for one week. You will be required to donate a small amount of blood to be retained by the Center. This sample will be used only as described for this study unless you provide additional consent (page 6). The sample will not have your name on it and will be frozen at the Center. If you do not provide additional consent, the unused sample will be destroyed at the end of the study. This blood sample will be drawn so we can test for some genes that may be related to how much you like exercise. A trained person will perform your blood draw to collect 3 to 5 milliliters, or about one teaspoon. This is much less than the pint or 475 milliliters blood banks draw every 8 weeks. We will test for genes related to brain dopamine, serotonin, and adrenaline. No individual information regarding genotypes will be made available to you or to a third party. This genotyping carries no medical or therapeutic value. There is no medical significance associated with the DNA test results. The sample will not be sold to others. You will then be scheduled for your 3 baseline visits.

Baseline Measurements:
A variety of measurements will be completed during the 3 baseline visits:

- Visit 1 (you must arrive 2-3 hours after eating):
  - o You will answer questions and do computer activities to see how much you like and value physical activity (60 min).

    - Using a computer, you will report everything you ate for 2 weekdays and 1 weekend day (30 minutes each day).

- Visit 2 we will measure (you must arrive at least 2 hours after eating):
    - Your body fat using a very low-dose x-ray machine (15 min)
    - Height and weight using a scale (1 min).
    - You will complete a moderate to hard intensity fitness test while walking on a treadmill. You will wear a tight-fitting face mask during the test, in addition to an activity monitor and/or chest strap to measure energy expended (60 min including rest and exercise time).

- Visit 3 (you must arrive at least 2 hours after eating):
    - You will be guided through a 15-minute exercise session on a treadmill and complete questions before, during, and after exercising (45 min).

Treatment Procedure:
You will be randomly assigned to one of two exercise groups during this study or to the control group. You will not have a choice as to which group you are in. Access to Choice Health and Fitness will be provided to you at no cost during the study. One exercise group will use 300 calories per exercise session and the other group will use 150 calories. This exercise will take about 30 to 60 minutes to complete. Most of the exercise sessions will be on your own schedule. The first 3-5 sessions will be supervised by trained research staff to introduce you to the equipment and instruct you on the intensity and length of exercise needed to reach your goal. Then, sessions will be supervised as needed to encourage you to follow the program. If you miss a session, you will have to make it up on another day. You will be asked to follow our exercise program for 6 weeks. For the four weeks after that you can exercise however you want. The control group will receive the exercise program and membership to Choice Health and Fitness after completing the 10 week study. During the first 6 weeks, you will complete the following measurements:

- Energy used during exercise - during all exercise sessions you will wear an activity monitor and/or chest strap to measure the energy used during exercise. The monitors transmit to a small display that you will use to make sure that you exercising at your prescribed intensity and amount.
- After 6 weeks: you will follow the baseline assessment procedure and schedule.
- One month after the end of the intervention (week 10) you will follow the baseline assessment schedule.

WHAT ARE THE RISKS OF THE STUDY?

There may be some risk from being in this study.

Questionnaires: You may feel upset when answering survey questions. Some questions may be sensitive, and you may become upset as a result. If, however, you become upset by any question, you may stop at any time or choose not to answer.

Blood sampling: There is a small risk of bruising or swelling from blood sampling. There may be pain as the needle passes through the skin. You may feel lightheaded or faint during or right after a blood draw. This is more likely to happen if you have had problems with fainting during blood draws in the past. To reduce these problems, trained and skilled personnel will ask you about your blood draw history. They will follow standard medical precautions to reduce risk. Please tell them if you have had problems in the past. No more than 5 ml (about 1 teaspoon) will be drawn during visit one so we can assess your genes. We will test for genes related to brain dopamine, serotonin, and adrenaline. No individual information regarding genotypes will be made available to you or to a third party. This genotyping carries no medical or therapeutic value. There is no medical significance associated with the DNA test results. The sample will not be sold to others.

Exercise and Fitness Tests: There is a small risk of sprains, strains, and broken bones. To reduce this risk, you will be watched closely by a scientist. Some soreness may occur 1-2 days after exercise but this will go away with time. Exercise can uncover or worsen hidden heart problems. It is unlikely you will have problems with your heart. If we see symptoms of any medical problems, testing will be stopped.

DXA Scan: The DXA scan is an x-ray and is considered to be a no greater than minimal risk method. The radiation dose of the whole-body scan is no more than 1.0 millirem. This dose is equivalent to approximately 1/620 of normal yearly background radiation, ¼ of the radiation received in a long flight, or 1/10 of the radiation received in a chest x-ray. A quality assurance check will be completed on the DXA each day prior to its use; the software will not allow the use of the DXA if the quality assurance check fails. Each subject will receive two DXA scans, and an extra scan may be needed. The effects of small doses of radiation on a developing fetus are not known; therefore, we will not knowingly allow a pregnant woman to have a DXA. Pregnancy tests will be done before the DXA if you are a woman of child-bearing age.

Armbands and chest straps: There is a possibility of skin irritation from the armband and the chest strap. These devices will be cleaned thoroughly between uses, and trained staff will instruct you on how to properly wear the devices. If any discomfort occurs, please report it to the research staff immediately and you will be instructed to remove the device or adjust its position.

Approval Date: JAN 6 2017
Expiration Date: JAN 5 2018
University of North Dakota IRB

Date _______
Subject Initials _______

WHAT ARE THE BENEFITS OF THIS STUDY?

We hope that, in the future, other people might benefit from this study as we hope to gain insight into what causes people to change their liking of an exercise program. This knowledge could improve weight-loss efforts in the future. You will get the chance to exercise under the supervision of a personal trainer and learn what types of exercise you prefer. You will not be given your measurements from this study.

WILL IT COST ME ANYTHING TO BE IN THIS STUDY?

You will not have any costs for being in this research study.

WILL I BE PAID FOR PARTICIPATING?

You will be paid for being in this research study. You may choose to receive a total of $500, or a 12-month individual membership to Choice Health & Fitness Center, or a 9-month family membership to Choice Health & Fitness Center for completion of the study. There is no payment for the screening visit. You will receive $150 for completing the baseline measures, $50 for completing the six weeks of exercise, and $300 for completing the end of the exercise training and testing visits. You will be required to provide your SSN and address to receive your payment check (W9 form).

WHO IS FUNDING THE STUDY?

There will be no independent funding for this research study. The USDA ARS-GFHNRC will provide funds to support the activities that are required to conduct the study. No one on the research team will receive a direct payment or an increase in salary for conducting this study.

CONFIDENTIALITY

The records of this study will be kept private to the extent permitted by law. In any report about this study that might be published, you will not be identified. None of the study results will have any names attached. Confidentiality will be maintained by assigning an identification number which will be used to namelessly code your research data for computer entry. This consent and the check information will be kept in a locked file at the Grand Forks Human Nutrition Research Center. Dr. James Roemmich and the staff assigned to the research study will have access to the data. Confidential information may be made available to the US Department of Agriculture as

Approval Date: JAN 6 2017
Expiration Date: JAN 5 2018
University of North Dakota IRB

Date ______
Subject Initials ______

specified in the USDA/ARS Privacy Act System of Records and to the University of North Dakota and as required by law or court order. Clinical trial information will be submitted to the National Institutes of Health/National Library of Medicine to be included in the clinical trials registry data bank (www.clinicaltrials.gov)

We will monitor you by video camera while you complete the computer activities used to determine how much you are motivated for physical activity, but you will not be recorded at any time during the study.

COMPENSATION FOR INJURY

In the event that this research activity results in an injury, treatment will be available including first aid, emergency treatment and follow-up care as needed. Payment for any such treatment is to be provided by you (you will be billed) or your third-party payer, if any (such as health insurance, Medicare, etc.) No funds have been set aside to compensate you in the event of injury. Also, the study staff cannot be responsible if you knowingly and willingly disregard the directions they give you.

IS THIS STUDY VOLUNTARY?

Your participation is voluntary. You may choose not to participate or you may discontinue your participation at any time without penalty or loss of benefits to which you are otherwise entitled. Your decision whether or not to participate will not affect your current or future relations with the University of North Dakota, Choice Health and Fitness, or the Grand Forks Human Nutrition Research Center.

If you decide to leave the study early, we ask that you call Bill Siders at 701-795-8430 or Kyle Flack at 701-795-8342 to inform them of your withdrawal.

You will be informed by the research investigator of this study of any significant new findings that develop during the study which may influence your willingness to continue to participate in the study.

There may be special circumstances that will result in your early withdrawal from the study without your approval. Such circumstances include realization that exercise is not healthy for you or realization that other conditions might make continued participation harmful to you. You will also be withdrawn from the study if you are not willing or not able to follow the study directions.

SUPPLEMENTAL INFORMATION ABOUT SAMPLES

Science and technology are advancing very rapidly. There may be additional research possible with this study. Part of this specific study is taking blood samples to be stored for future studies of activity behaviors.

You are being asked for your permission to let us keep some of the samples that are leftover and use them for future studies. You will not be contacted about any potential use of these samples. The samples will be kept indefinitely. The samples will be stored separately from this consent and there will be no link to any of your personally identifiable information. Despite efforts to keep your personal information confidential, absolute confidentiality cannot be guaranteed. Confidential information may be made available to the US Department of Agriculture as specified in the USDA/ARS Privacy Act System of Records and to the University of North Dakota and as required by law or court order. Clinical trial information will be submitted to the National Institutes of Health/National Library of Medicine to be included in the clinical trial registry data bank (www.clinicaltrials.gov).

Please indicate below if you consent that your samples may be used in future research. You will not be paid an additional amount for this consent, and this is not required for participation in the current study. If you choose not to allow the use of your sample for future research your sample will be destroyed at the end of the study.

(Please circle one) Yes No

Initials ________

CONTACTS AND QUESTIONS?

The researchers leading this study are Dr. James Roemmich and Dr. Kyle Flack. You may ask any questions you have now. If you later have questions, concerns, or complaints about the research please contact Dr. Flack at 701-795-8342 or Bill Siders at 701-795-8430 during the day or leave a message at 701-795-8488 after hours.

If you have questions about your rights as a research subject, or if you have any concerns or complaints about the research, you may contact the University of North Dakota Institutional Review Board at (701) 777-4279. Please call this number if you cannot reach research staff, or you wish to talk with someone else. General information about being a research subject can be found by going online and clicking "Information for Research Participants" on the web site: http://und.edu/research/resources/human-subjects/resarch-participants.cfm

Approval Date: JAN 6 2017
Expiration Date: JAN 5 2018
University of North Dakota IRB

Your signature indicates that this research study has been explained to you, that your questions have been answered, and that you agree to take part in this study. You will receive a copy of this form.

Subjects Name: ____

______________________________ ________________
Signature of Subject Date

I have discussed the above points with the subject or, where appropriate, with the subject's legally authorized representative.

______________________________ ________________
Signature of Person Who Obtained Consent Date

Approval Date: JAN 6 2017
Expiration Date: JAN 5 2018
University of North Dakota IRB

Date ______
Subject Initials ______

Timeline

Screening Visit:
- Complete forms to determine eligibility
- Sign informed consent
- Complete exercise questionnaires
- Be given an activity monitor
- Provide a blood sample

Visit Two:
- Measure body weight, height, and body fat.
- Complete a moderate to hard intensity fitness test while walking on a treadmill.

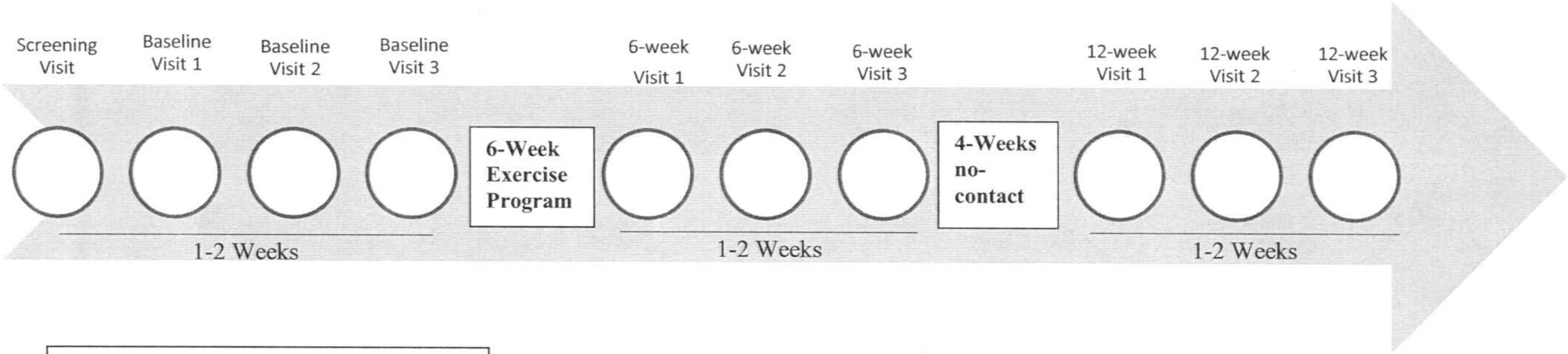

Visit one:
- Complete questionnaires and computer activities to determine how much you like and value physical activity.
- Exercise under the guidance of a personal trainer

Visit Three:
- You will be guided through a 15-minute exercise session on a treadmill
- Complete questionnaires before, during, and after exercising

Approval Date: JAN 6 2017
Expiration Date: JAN 5 2018
University of North Dakota IRB

Date ______
Subject Initials ______

ADDENDUM TO CONSENT

TITLE: The Motivation to Exercise

If you have already completed the following tests for the Exercise Genes Study, we will use that data for this study instead of testing you again.

1. Wearing of an activity monitor for one week.
2. Blood draw to test for some genes that may be related to how much you like exercise.
3. Answering questions and doing computer activities to see how much you like and value physical activity.
4. Measuring your body fat using a very low-dose x-ray (dual-energy x-ray absorptiometry [DXA]) machine.

Your signature indicates that this addendum has been explained to you, that your questions have been answered, and that you agree to have your data from the Exercise Genes Study used for the Motivation to Exercise Study instead of retesting. You will receive a copy of this form.

Subjects Name: ______________________________

______________________________ ____________
Signature of Subject Date

I have discussed the above points with the subject or, where appropriate, with the subject's legally authorized representative.

______________________________ ____________
Signature of Person Who Obtained Consent Date

Approval Date: JAN 6 2017
Expiration Date: JAN 5 2018
University of North Dakota IRB

Date ______
Subject Initials ______
